# High-throughput imaging-based nephrotoxicity prediction for xenobiotics with diverse chemical structures

**DOI:** 10.1007/s00204-015-1638-y

**Published:** 2015-11-27

**Authors:** Ran Su, Sijing Xiong, Daniele Zink, Lit-Hsin Loo

**Affiliations:** 1 Bioinformatics Institute, 30 Biopolis Street, #07-01 Matrix, Singapore, 138671 Singapore; 2 Institute of Bioengineering and Nanotechnology, 31 Biopolis Way, The Nanos, Singapore, 138669 Singapore; 3 Department of Pharmacology, Yong Loo Lin School of Medicine, National University of Singapore, 10 Medical Drive, Singapore, 117597 Singapore

**Keywords:** Nephrotoxicity, Toxicity prediction, Phenotypic profiling, High-content screening, In vitro model, DNA damage response

## Abstract

The kidney is a major target for xenobiotics, which include drugs, industrial chemicals, environmental toxicants and other compounds. Accurate methods for screening large numbers of potentially nephrotoxic xenobiotics with diverse chemical structures are currently not available. Here, we describe an approach for nephrotoxicity prediction that combines high-throughput imaging of cultured human renal proximal tubular cells (PTCs), quantitative phenotypic profiling, and machine learning methods. We automatically quantified 129 image-based phenotypic features, and identified chromatin and cytoskeletal features that can predict the human in vivo PTC toxicity of 44 reference compounds with ~82 % (primary PTCs) or 89 % (immortalized PTCs) test balanced accuracies. Surprisingly, our results also revealed that a DNA damage response is commonly induced by different PTC toxicants that have diverse chemical structures and injury mechanisms. Together, our results show that human nephrotoxicity can be predicted with high efficiency and accuracy by combining cell-based and computational methods that are suitable for automation.

**Electronic supplementary material:**

The online version of this article (doi:10.1007/s00204-015-1638-y) contains supplementary material, which is available to authorized users.

## Introduction

The kidney plays an important role in the filtration and active elimination of xenobiotics from the plasma (Tiong et al. 2014). Foreign compounds originating from medicine, food, or the environment are actively transported and metabolized by the renal proximal tubular cells (PTCs; Commandeur and Vermeulen 1990). After uptake, xenobiotics and their metabolites/intermediates may damage the PTCs (Townsend et al. 2003; Stiborová et al. 2003) and lead to acute kidney injury or chronic kidney disease (Choudhury and Ahmed 2006; Tiong et al. 2014). Therefore, accurate methods for predicting nephrotoxicity are critical for the safety assessment of xenobiotics, and the management of the health and environmental hazards posed by these compounds.

There are several existing approaches for predicting xenobiotic toxicity in human. Animal testing is a standard approach, but suffers from the problems of long turnaround time, low throughput, and sometimes poor prediction of human toxicity (Krewski et al. 2010). This approach is especially unsuitable for evaluating the large numbers of existing and ever-increasing numbers of novel synthetic compounds, such as chemicals and nanoparticles. In fact, the current interest in alternatives to animal testing is driven by the requirement for efficient testing of large numbers of compounds with diverse chemical structures and injury mechanisms. This is, for instance, reflected by current legislations, such as the regulation on “Registration, Evaluation, Authorization and restriction of CHemicals” (REACH) in the European Union (Lilienblum et al. 2008). Computational modeling of quantitative structure–activity relationships (QSAR) is a rapid approach and works well for compounds with specific or well-understood chemical structures or mechanisms (Cherkasov et al. 2014). However, most QSAR models do not consider the biological contexts of compound exposure and therefore have limited applications in predicting the complex biological responses, such as organ-specific toxicity, of compounds with diverse chemical structures. Finally, in vitro assays based on immortalized, primary, or stem-cell-derived renal cells may provide a balance between throughput and physiological relevance. However, most of the current cell-based assays were either tested with very small numbers of nephrotoxicants (usually <5; Jang et al. 2013; Tiong et al. 2014), or poorly predictive of organ-specific toxicity in large-scale studies (Lin and Will 2012). Therefore, accurate prediction of nephrotoxicity remains challenging, and there is currently no regulatory approved in vitro test for nephrotoxicity.

Recently, we have developed nephrotoxicity models based on compound-induced interleukin (IL)-6/8 expression levels in immortalized and primary human PTCs (Li et al. 2013; Su et al. 2014), human embryonic stem cell- (Li et al. 2014), and induced pluripotent stem cell-derived PTC-like cells (Kandasamy et al. 2015). We rigorously evaluated the performance of these models using a large set (~30–40) of structurally diverse compounds, which included non-PTC-toxic nephrotoxicants and non-nephrotoxic compounds as negative reference compounds. Due to the relatively high test accuracies of these models (~75.3 %), we hypothesize that there may be PTC-specific injuries that are commonly induced by PTC toxicants with diverse structures and targets. Furthermore, the RNA isolation and qPCR steps of the IL-6/8 measurements are difficult and costly to be automated for high-throughput applications. Therefore, there is still a need to develop an alternative high-throughput, cost-effective, and accurate nephrotoxicity prediction approach, which may also provide new insights into the cell injuries and responses induced by these compounds.

Xenobiotic-induced injuries impair cellular functions and lead to changes in cellular phenotypes, such as reorganizations and changes of cellular and subcellular structures. One of the main advantages of predicting toxicity based on cellular phenotypes is that the cell injury mechanisms do not need to be defined a priori. This is especially useful for building models for a diverse set of xenobiotic compounds that may induce the same types of injury and responses, but through different biochemical mechanisms. Models based on specific mechanisms may only cover specific classes of compounds, and not be generally applicable to other compounds (Tiong et al. 2014). Quantitative image-based profiling of cellular phenotypes under large numbers of compounds has become feasible due to the advances in automated microscopy and image processing methods (Feng et al. 2009). Loo and colleagues have previously developed computational methods to automatically construct phenotypic profiles from large numbers of unbiased and quantitative descriptors (or features) of cellular phenotypes based on microscopy images of cells (Loo et al. 2007; Laksameethanasan et al. 2013). These profiles were used to distinguish large numbers of compounds with different targets/mechanisms (Loo et al. 2007) or proteins involved in different biological processes (Loo et al. 2014). Here, we present a study that uses similar phenotypic profiling methods to screen for a compact set of phenotypic features that are predictive of in vivo PTC toxicity of xenobiotic compounds. Our approach for phenotypic profiling is conceptually similar to the approach of using gene expression profiling to identify differentially expressed genes, except that we screen for phenotypic changes, which are more likely to be commonly induced by toxicants with different mechanisms; and we can test much larger numbers of compounds than RNA sequencing or microarray assays (Feng et al. 2009).

## Results

### Reference compound list

**Fig. 1:**
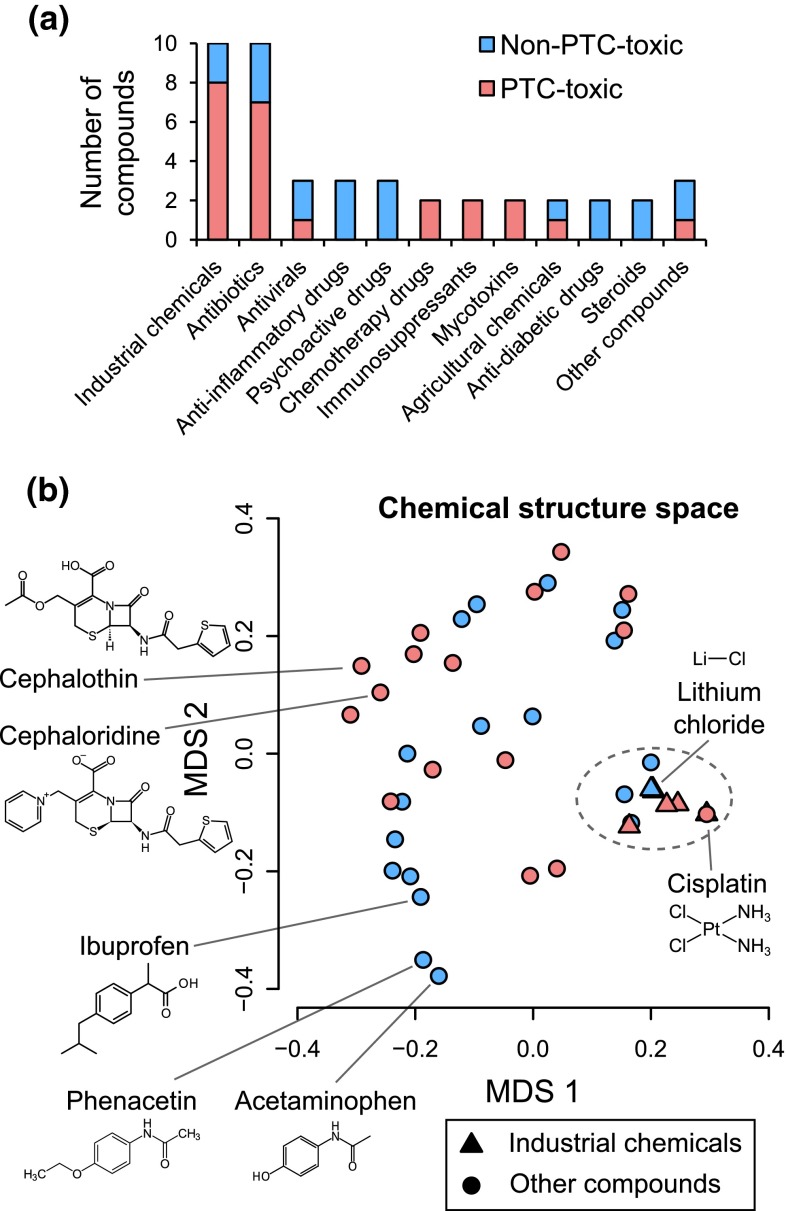
Reference xenobiotic compounds in our study have diverse chemical structures. **a** Categorization of the reference xenobiotic compounds used in our study according to their sources or applications. **b** Multi-dimensional scaling plot showing the chemical structure dissimilarity based on Tanimoto coefficients between all the reference compounds (MDS1/2 = the first and second coordinates of the multi-dimensional scaling, *dashed line* = a cluster of compounds with simple and similar chemical structures. All industrial chemicals are grouped into this cluster together with other compounds irrespective of their known PTC toxicity. Many compounds within the cluster are on *top* of each other.)

To make our computational models more comprehensive, we increased the number of reference xenobiotic compounds to 44 (Supplementary Material 1—Table S1), among which 38 compounds were previously used in our IL-6/8-based models (Li et al. 2013, 2014; Su et al. 2014; Kandasamy et al. 2015). These reference compounds included commonly used industrial chemicals, antibiotics, antivirals, chemotherapy drugs, mycotoxins, agricultural chemicals and other compounds (Fig. 1a). They were divided into two groups based on their known in vivo toxicity from published clinical and/or animal studies [detailed information for most of the compounds can be found in our previous reports (Li et al. 2014; Kandasamy et al. 2015)]. The “PTC-toxic” group had 24 nephrotoxicants known to damage PTCs, and the “non-PTC-toxic” group had 14 nephrotoxicants not known to damage PTCs in humans and 8 compounds not known to damage the human kidney. Furthermore, ten and eight compounds in the PTC-toxic and non-PTC-toxic groups, respectively, were known to be hepatotoxic (Supplementary Material 1—Table S1). This design of reference compound list ensured that we would not favor phenotypic features for general or non-PTC-specific toxicity. Our binary categorization of the compounds simplified the prediction problem and allowed us to use well-established prediction performance criteria to evaluate different phenotypic features and cell types. Our reference compounds had diverse chemical structures, and we found no obvious structural difference between the PTC-toxic and non-PTC-toxic compounds (Fig. 1b). For example, all of the industrial chemicals and a few other compounds were clustered together in the chemical space irrespective of their known PTC toxicity (dashed line in Fig. 1b). Most of the compounds within the cluster were metallic compounds, such as cisplatin (PTC-toxic) and lithium chloride (non-PTC-toxic), which have very simple and thus hard-to-differentiate molecular structures. We treated primary human PTCs from three different donors with these compounds in seven different doses (1.6–1000 μg/mL) for 16 h.

### Automated cellular phenotypic profiling

**Fig. 2:**
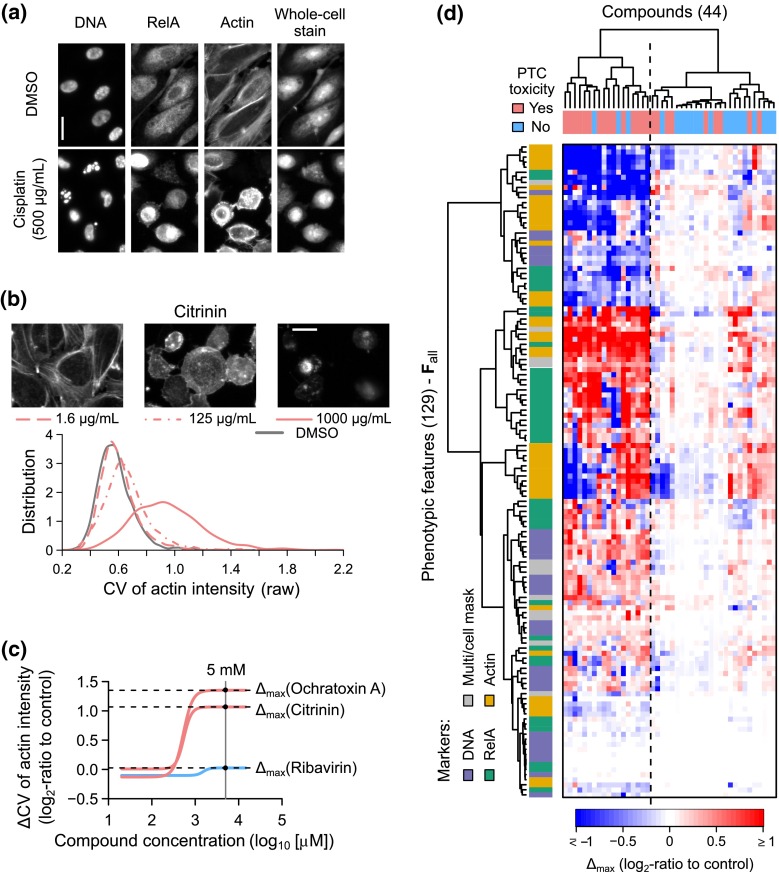
Quantitative image-based phenotypic profiles of primary human proximal tubule cells treated with the reference compounds. **a** Immunofluorescence images showing the four fluorescence markers used in the HPTC-A dataset for primary PTCs treated with the DMSO control or 500 µg/mL cisplatin (*scale bar* 20 µm). **b** Single-cell probability distribution functions for the raw coefficient of variation (CV) of actin intensity values measured from primary PTCs treated with different concentrations of citrinin (*light red lines*) or DMSO (*gray line*). Exemplary fluorescent images for the actin stains are shown above the distribution function plots (*scale bar* 20 µm). **c** Concentration response curves for changes in the CV of actin intensity induced by three of the reference compounds (*light red* = PTC-toxic compounds, *light blue* = non-PTC-toxic compound). The maximum response value ($\varDelta_{\hbox{max} }$) for each compound was determined from its response curve at 5 mM. **d** Heatmap showing the $\varDelta_{\hbox{max} }$ values for all the 129 phenotypic features (*rows*) measured from primary human PTCs treated with 44 reference compounds (*columns*). (*Dendrograms* = hierarchical clustering of the compounds or features based on the $\varDelta_{\hbox{max} }$ values, *dash line* = separation between the two major clusters identified from the clustering of compounds)

Our phenotypic profiling strategy (Supplementary Material 1—Fig. S1) was to automatically measure a large number of quantitative phenotypic features of primary human PTCs under in vitro conditions, and then systematically screen for a subset of phenotypic features that were the most predictive of in vivo PTC toxicity. Our features were based on four fluorescent markers (Fig. 2a). We used 4′,6-diamidino-2-phenylindole (DAPI) and rhodamine phalloidin to label the DNA and actin cytoskeleton, respectively. Nuclear and chromatin structure alterations are involved in many fundamental cellular processes, such as transcription, mitosis, and cell death. Actin filaments play an important role in maintaining the cellular function of PTCs (Kellerman et al. 1990). We also labeled the cells with an antibody specific for a subunit of the nuclear factor (NF)-κB complex, RelA. This was motivated by our previous models based on IL-6/8 (Li et al. 2013, 2014; Su et al. 2014; Kandasamy et al. 2015), which are regulated by the NF-κB complex (Matsusaka et al. 1993). The final marker was a whole-cell stain (WCS) used to facilitate automated cell segmentation and measurements of cellular morphology features.

After compound treatment, we stained PTCs with these four fluorescent markers and imaged them using a high-throughput imaging system. We automatically identified ~500–1000 cells from 36 microscopy images captured for each compound and treatment dosage (Supplementary Material 1—Fig. S2). Then, for each cell, we extracted 129 quantitative phenotypic features (Fig. 2b and Supplementary Material 2), which include 78 Haralick’s texture features (Haralick et al. 1973; measuring the statistics of the spatial co-occurrence patterns of the markers), 29 intensity features (measuring the staining levels of the markers at different subcellular regions), 9 intensity ratio features (measuring the ratios between intensity features), 6 correlation features (measuring the spatial correlations between two markers at the single-cell level), and 6 morphology features (measuring the shape properties of the nuclear and cellular regions). We also included cell count as a feature. Similar phenotypic features and profiling methods were previously used to classify large numbers of small molecules according to their targets/mechanisms (Loo et al. 2007). Therefore, we hypothesized that a subset of these features might also be discriminative enough to predict PTC toxicity.

For each phenotypic feature, we first computed the log_2_-ratios (“$\varDelta$”) of its values at all the tested dosages with respect to the vehicle controls. Then, we estimated the feature’s dose response curve using a standard log-logistic model, and its maximum response value (“$\varDelta_{\hbox{max} }$”) from the curve (Fig. 2c). Finally, the median $\varDelta_{\hbox{max} }$ values across three biological replicates were computed. The final dataset (“HPTC-A”) was a matrix of $\varDelta_{\hbox{max} }$ values for 129 phenotypic features (${\mathbf{F}}_{\text{all}} = \{ {\mathbf{f}}_{1} ,\;{\mathbf{f}}_{2} ,\; \ldots ,\;{\mathbf{f}}_{129} \}$, rows) and 44 xenobiotic compounds (columns; Fig. 2d and Supplementary Material 3). For brevity, all features that we mention in this article are referring to the $\varDelta_{\hbox{max} }$ values of the respective features and not the raw measured feature values, unless otherwise indicated. Hierarchical clustering of the compounds based on the phenotypic feature values revealed two major clusters (Fig. 2d). One of them was significantly enriched with the PTC-toxic compounds (83 % of the cluster were PTC-toxic compounds; *P* = 1.59 × 10^−3^, hypergeometric test), and the other one was significantly enriched with the non-PTC-toxic compounds (65 % of the cluster were non-PTC-toxic compounds; *P* = 1.59 × 10^−3^, hypergeometric test). Most of the phenotypic features showed larger changes under the first cluster than the second cluster, suggesting that non-PTC-toxic compounds only induced small or no change in the primary human PTCs. We also performed similar clustering analysis on the phenotypic features and found two major clusters corresponding to either increased or decreased feature values after treatments with PTC-toxic compounds (Fig. 2d). Features from all markers were represented in both clusters. Therefore, most of our phenotypic features are diverse and capture both increasing and decreasing properties of the markers.

### Nuclear and cytoskeletal features are highly predictive

**Fig. 3:**
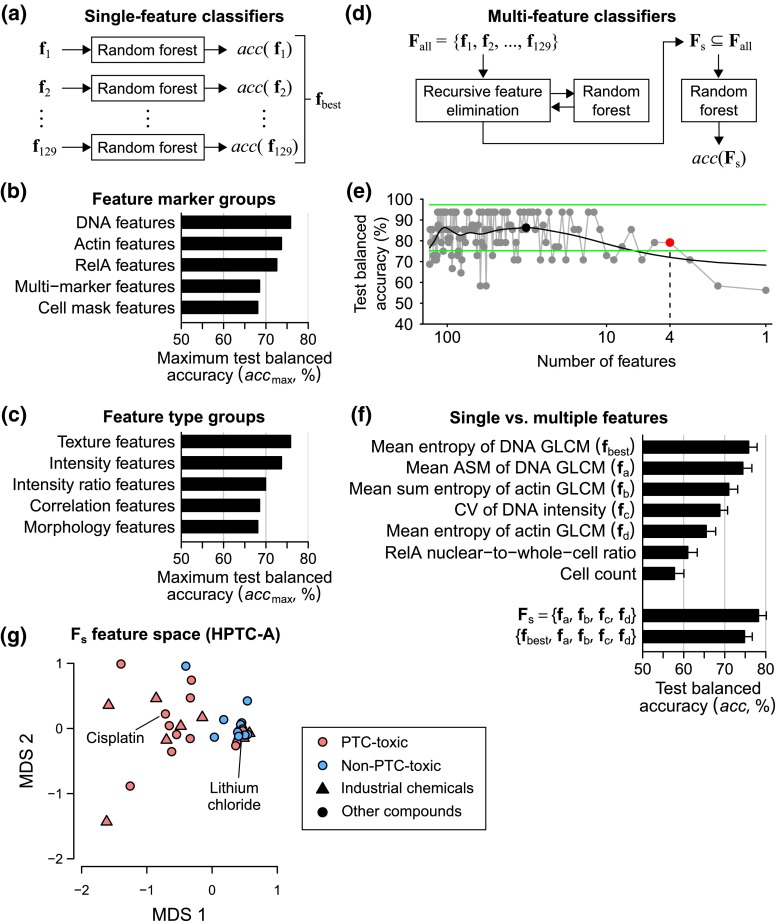
Human in vivo nephrotoxicity can be accurately predicted based on in vitro DNA and cytoskeleton features of PTCs. **a** Schematic showing the procedure for identifying the best single feature (${\mathbf{f}}_{\text{best}}$) from all the 129 phenotypic features. The test balanced accuracies of the classifiers based on the best single features from different **b** feature marker groups or **c** feature type groups in the HPTC-A dataset. **d** Schematic showing the procedure for identifying the best feature subset (${\mathbf{F}}_{s}$) from all the 129 phenotypic features. **e** An example of the output of automated feature elimination from one of the 10 cross-validation folds. The performance of the feature subset selected during each iteration of our recursive feature elimination algorithm is shown, starting from all features to the last retained feature (*gray dots* = test balance accuracy of the feature subset retained during each iteration, *black line* = spline interpolation of all the test balance accuracy values, *black dot* = local maximum with the smallest number of features, *green lines* = upper and lower 5-percentiles or limits of the Gaussian distribution centered around the last local maximum, *red dot* = the test balanced accuracy of the final selected feature subset, which is the subset with the smallest number of features and accuracy value between the *upper* and *lower* limits). In this example, the final selected number of features was four. Any further elimination of features would reduce the performance of the classifier. **f** Comparison of the test balanced accuracies among different single- and multi-feature classifiers for the HPTC-A dataset. The accuracy values were estimated using 10 × 10-fold cross-validations (*error bars* = standard errors of the means). **g** Multi-dimensional scaling plot showing the phenotypic dissimilarity between all the reference compounds in the HPTC-A dataset based on their Euclidean distances in ${\mathbf{F}}_{s}$ (MDS1/2 = the first and second coordinates of the multi-dimensional scaling)

To test each individual feature, we constructed a binary classifier based on the feature using a random forest algorithm (Breiman 2001; Su et al. 2014) and estimated the prediction accuracy using a tenfold cross-validation procedure (Fig. 3a, Methods, and Supplementary Material 1—Text S1). In theory, the balanced accuracy (average of sensitivity and specificity) of a binary classifier ranges from 50 % (performance of a trivial random classifier) to 100 % (maximum); but in practice, it may go slightly below 50 %. The training accuracy is the accuracy in classifying the training data used to build the classifier, and the test accuracy is the accuracy in classifying independent test data unused during the training process. We used all the compounds during cross-validation, but our evaluation procedure ensured that the training and test samples were coming from different compounds and statistically independent. We repeated the cross-validation procedure 10 times with different random fold divisions, and all the mean accuracy values presented in this report were obtained by averaging the accuracy values from every fold and trial.

For the HPTC-A dataset, we found that all single features had ~97 % and above training accuracy and ~43–76 % test accuracy (Supplementary Material 2). The high training-accuracy values do not imply all the features had similar or indistinguishable training performance, because the training accuracies of the features was still strongly correlated with their test accuracies (*r* = 0.842, Supplementary Material 1—Fig. S3). In our unbiased approach for phenotypic profiling, we started with a large number of general phenotypic features, but only expected a small number of features to be discriminative. We aimed to find out which of the four fluorescent markers and which of the five feature types produced the most discriminative features. Therefore, we grouped all the features according to their fluorescent markers or feature types and compared the maximum achievable accuracies of these feature groups. The feature marker group with the highest maximum test accuracy was DNA (75.8 %), followed by actin (73.7 %) and RelA (72.6 %; Fig. 3b). Surprisingly, RelA features were not highly predictive of PTC toxicity. For example, the RelA nuclear-to-whole-cell intensity ratio, which is an indicator of NF-κB nuclear translocation and transcriptional activation of its downstream effectors (Deptala et al. 1998), only had 61.0 % test accuracy. The feature type groups with the highest maximum test accuracy was Haralick’s texture (Haralick et al. 1973; 75.8 %), followed by intensity (73.7 %) and intensity ratio (69.9 %; Fig. 3c). In fact, six of the ten best-performing single features were all Haralick’s texture features, which are based on the gray-level co-occurrence matrices (GLCM; Haralick et al. 1973) of the fluorescent markers. The GLCM of a marker summarizes the distribution of spatial transitions between different intensity levels of the marker in a cell image (Haralick et al. 1973). Haralick’s features, which describe various statistical properties of a GLCM, can be used to represent the textural patterns found in the image (Methods). The best single feature, ${\mathbf{f}}_{\text{best}}$, among all the 129 features was the “mean entropy” of the DNA GLCM (75.8 % test accuracy). The feature is a measure of the homogeneity of the DNA GLCM. Cell images with more “random” DNA staining patterns, where the transitions between all intensity levels are more equally probable, have more homogenous GLCMs and thus higher values of GLCM entropy (Supplementary Material 1—Fig. S4). Overall, changes in the texture of the DNA and actin cytoskeleton localization patterns were highly predictive of the in vivo PTC toxicity of xenobiotics with diverse chemical structures. The high accuracy underscores the importance and advantage of using image-based phenotypic features as in vitro toxicity endpoints.

### Multiple features are more predictive than single features

Xenobiotic compounds may induce different types of PTC injuries and responses. Therefore, classifiers based on multiple different phenotypic endpoints are more likely to give higher overall prediction accuracy. To preserve the dependency between features, we trained multi-dimensional classifiers based on multiple features simultaneously (Fig. 3d). Then, a recursive feature elimination algorithm (Loo et al. 2007) was used to automatically remove irrelevant and/or redundant features (Fig. 3e, Methods, and Supplementary Material 1—Text S1). The number of retained features was automatically determined based on the training data only. Therefore, the process was repeated for every cross-validation fold, which had different training data. The features were ranked according to their importance values, which were estimated by the random forest algorithm (Breiman 2001) and averaged across all the cross-validation folds. For the HPTC-A dataset, we found a set of four features (${\mathbf{F}}_{s}$) that had the highest average importance values (Supplementary Material 1—Fig. S5). These features were the “coefficient of variation (CV)” of DNA intensity at the nuclear region, “mean angular second moment (ASM)” of the DNA GLCM, “mean sum of entropy” and “mean entropy” of the actin GLCM (Fig. 3f and Supplementary Material 1—Table S2). Similar to the single-feature classification results, these top features were all based on the DNA and cytoskeleton markers, and three of them were texture features. We trained multi-feature classifiers using these four features and obtained 78.3 % test accuracy, which were higher than the performances of all single-feature classifiers (Fig. 3f). The individual test accuracies of these four features only ranged between 65.5 and 74.4 %. Therefore, combining the features together increased the prediction performance. We also found that the inclusion of ${\mathbf{f}}_{\text{best}}$ into ${\mathbf{F}}_{s}$ did not further increase the prediction accuracy of our models (Fig. 3f), indicating our recursive feature elimination algorithm was highly effective.

In the feature space of ${\mathbf{F}}_{s}$, we found that most of the non-toxic compounds formed a tight cluster, where they were closer to each other than to the toxic compounds (Fig. 3g). Six of the eight toxic industrial chemicals with simple chemical structures could now be clearly separated from the non-toxic compounds (Fig. 3g). This separation was not evident in the original chemical structure space (Fig. 1b). Among all the tested compounds, 22 compounds were consistently being correctly classified by both the single- and multi-feature classifiers (Supplementary Material 1—Table S3). Only three compounds, namely ciprofloxacin (antibiotic), levodopa (psychoactive drug), and copper(II) chloride (industrial chemical), had consistently <50 % test accuracy in both types of classifiers. These results show that our computational models were general and did not favor specific classes of compounds.

### The most important feature indicates induction of a DNA damage response

**Fig. 4:**
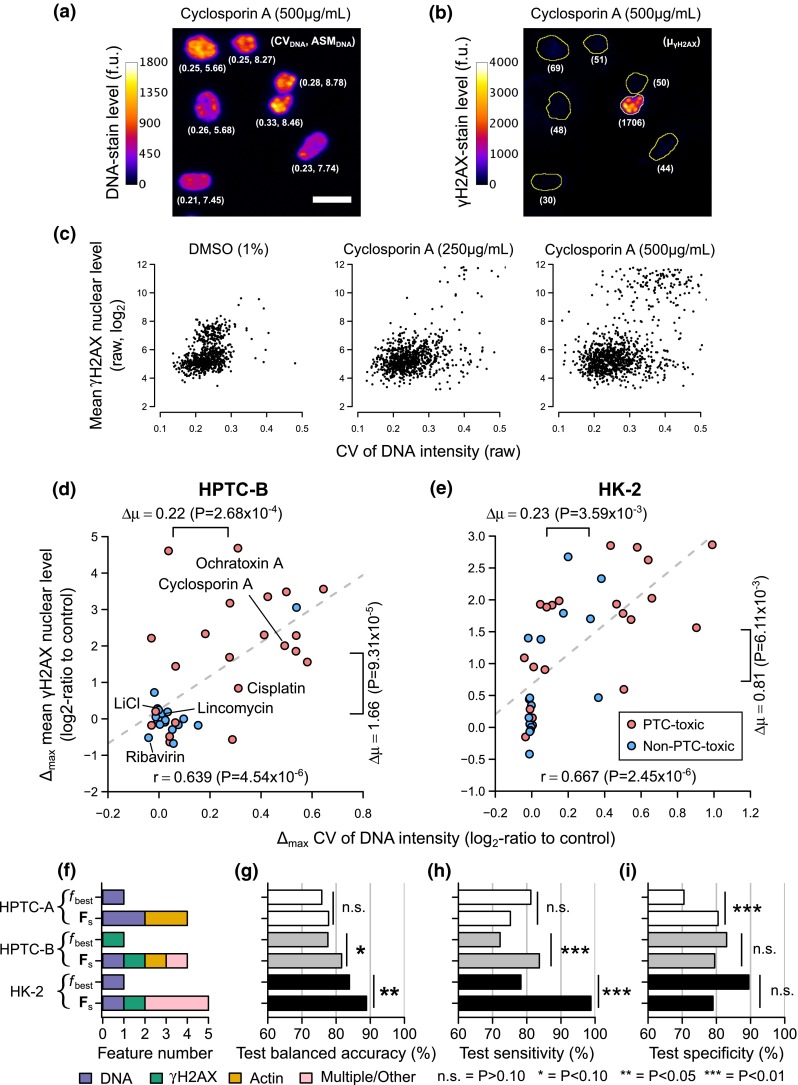
Most PTC toxicants induce a DNA damage response under in vitro conditions. Exemplary immunofluorescence images from the HPTC-B dataset showing the **a** DNA and **b** γH2AX staining levels of primary human PTCs treated with cyclosporine A (*yellow lines* = automatically determined nuclear boundaries, *scale bar* 20 µm). The quantified values for the CV of DNA, ASM of DNA GLCM, and mean nuclear γH2AX level are shown in the *parentheses below the cells*. **c** Scatter plots showing the raw CV of DNA and mean nuclear γH2AX level of primary PTCs treated with different dosages of cyclosporine A or DMSO (*dots* = single-cell measurements quantified from the images). Scatter plots showing the maximum responses ($\varDelta_{\hbox{max} }$) in the CV of DNA and mean nuclear γH2AX level for the **d** HPTC-B or **e** HK-2 datasets (*circles* = compounds, $\varDelta \mu$ = difference between the mean values of PTC-toxic and non-PTC-toxic compounds, *dashed lines* = optimum linear-regression fits of the data, *r* = Pearson’s correlation coefficient; all *P* values shown were obtained from one-sided *t* tests). The six compounds selected for studying the relationships between the DNA damage response and cell death are highlighted. The **f** distribution of markers, **g** test balanced accuracy, **h** test sensitivity, and **i** test specificity of the best single and multiple features for all three datasets (all *P* values were obtained using a one-sided Wilcoxon signed-rank test)

To further investigate the type of cell injury and damage response represented by our phenotypic features, we focused on the two DNA features in ${\mathbf{F}}_{s}$, namely 1) the mean ASM of the DNA GLCM, which had the highest single-feature test accuracy among the four selected features, and 2) the CV of DNA intensity at the nuclear region (Fig. 3f). ASM is a measure of the heterogeneity of a DNA GLCM (Methods and Fig. 4a). The feature gives high values when the transitions between certain intensity levels are dominant (for example, when the intensity values form certain regular shapes), or low values when all transitions are equally probable (for example, when the intensity values are diffused and randomly distributed). CV, which is equal to standard deviation divided by mean, is a standardized measure of the dispersion of a set of values, which in our case were the DNA staining intensity levels within the nuclear region. By examining the fluorescence microscopy images of the cells, we found that cells with high values of these two features had disconnected, highly irregular, and punctate DNA staining levels (Fig. 4a), indicating profound changes in chromatin structure and the formation of distinct chromatin domains. However, the overall nuclear and cellular morphologies of these cells remained largely intact and not fragmented, as in typical apoptotic cells. Therefore, we hypothesized that the features may indicate a DNA damage response, which is known to be associated with the formation of distinct chromatin domains in the megabase size range and large-scale chromatin reorganization (Rogakou et al. 1998; Jakob et al. 2011).

To test our hypothesis, we repeated the treatment experiments for 42 reference compounds, but replacing the RelA marker with an antibody specific for histone H2AX phosphorylated on serine 139 (γH2AX), which is a DNA damage response marker (Rogakou et al. 1998; Fig. 4b). Under endogenous or exogenous DNA damage conditions, γH2AX is induced and recruits repair factors to the sites of double-strand breaks (Paull et al. 2000). We repeated the experiments in both primary human PTCs (the “HPTC-B” dataset) and an immortalized human PT cell line, human kidney 2 (the “HK-2” dataset, Supplementary Material 1—Fig. S6). At the single-cell level, we found that cells with higher raw DNA CV levels induced by xenobiotic compounds tended to have higher raw mean γH2AX nuclear levels, but the responses might be highly heterogeneous (Fig. 4b). For example, 500 μg/mL cyclosporin A caused ~40-fold increases in the raw mean γH2AX nuclear levels, but only in ~13 % of the cells (Fig. 4c). Nevertheless, due to the large increases in magnitude, the effects could still be detected at the population-averaged level. Similar increases in γH2AX nuclear levels were also observed in cells treated with other PTC-toxic compounds (Supplementary Material 1—Fig. S6). Across all the tested compounds, the maximum increases (i.e., $\varDelta_{\hbox{max} }$) in DNA CV and mean nuclear γH2AX levels were strongly and positively correlated with each other in both primary and HK-2 cells (*r* = 0.639 and 0.667, respectively; Fig. 4d, e). Furthermore, both features were significantly higher in cells treated with the PTC-toxic compounds than those with the non-PTC-toxic compounds (all *P* < 0.01, one-tailed *t* test; Fig. 4d, e). These results suggest that most of the PTC-toxic compounds induce a DNA damage response, even though many of them are not known to bind to DNA directly.

### Improved computational models based on γH2AX

To what extent can the γH2AX marker improve the prediction performance of our computational models? We repeated the same phenotypic profiling procedure but using 129 phenotypic features based on the DNA, γH2AX and actin markers (Fig. 4f, g). For the HPTC-B dataset, we found that the best single feature ${\mathbf{f}}_{\text{best}}$ was the ratio of total γH2AX levels at the nuclear to the whole-cell regions (77.6 % test accuracy), which indicates the generation of γH2AX at the nuclear region (Supplementary Material 1—Fig. S4). The best multi-feature set ${\mathbf{F}}_{s}$ were four nuclear and actin cytoskeletal features (81.6 % test accuracy, see Supplementary Material 1—Fig. S5 and Table S2 for the complete listing of features). For the HK-2 dataset, we found that its ${\mathbf{f}}_{\text{best}}$ was the mean correlation of DNA GLCM (83.9 % test accuracy), which is a measure of the linear dependency of intensity levels of neighboring pixels (Supplementary Material 1—Fig. S4). The best multi-feature set ${\mathbf{F}}_{s}$ were five chromatin and cytoskeleton features (88.9 % test accuracy, Supplementary Material 1—Fig. S5 and Table S2). For both datasets (HPTC-B and HK-2), we found a consistent trend that multi-feature classifiers had significantly higher test accuracies than single-feature classifiers (Fig. 4f, g, *P* = 0.075 and 0.024, one-sided Wilcoxon signed-rank test). However, single-feature classifiers had higher test specificities, while multi-feature classifiers had higher sensitivities (Fig. 4h, i). Furthermore, the number of compounds that could be predicted with 100 % average test accuracy in both single- and multi-feature classifiers had increased from 22 (HPTC-A) to 25 (HPTC-B) or 28 (HK-2) (Supplementary Material 1—Table S3). Together, these results show that the inclusion of the γH2AX marker allowed us to obtain higher prediction accuracies.

We also compared the optimum phenotypic features selected for all three datasets (Supplementary Material 1—Table S2) and found several interesting and consistent trends. First, the mean ASM of DNA GLCM was automatically selected in the ${\mathbf{F}}_{s}$’s for both HPTC-A and HPTC-B datasets. Second, the ${\mathbf{f}}_{\text{best}}$ for the HPTC-B dataset (i.e., ratio of total γH2AX levels at the nuclear to the whole-cell regions) and one of the features in the ${\mathbf{F}}_{s}$ for the HK-2 dataset (i.e., ratio of total γH2AX and DNA intensity levels at the whole-cell region) are two closely related features that indicate nuclear increase in γH2AX levels (Supplementary Material 1—Fig. S4). Third, although the best single features were based on DNA or γH2AX markers, actin features were still retained by the multi-feature classifiers, suggesting that the actin marker was needed to correctly classify some compounds that induced actin remodeling. Together, these results show that our predictive models are highly reproducible, and a xenobiotic-induced DNA damage response is a general phenomenon that occurs in both human primary and immortalized PTCs.

### Cell death responses are variable

**Fig. 5:**
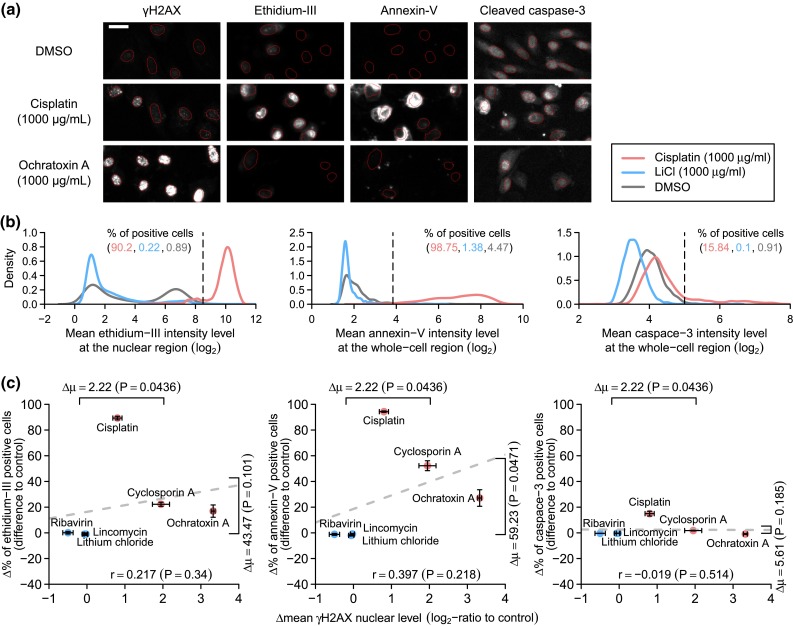
PTC toxicants induce variable cell death responses. **a** Immunofluorescence images showing the γH2AX, ethidium homodimer III, annexin-V, and cleaved caspase-3 staining levels of primary human PTCs treated with DMSO, cisplatin, and ochratoxin A (*red lines* = automatically detected nuclear boundaries, *scale bar* 20 µm). **b** Probability density function plots showing how the thresholds (*vertical dashed lines*) for ethidium-III-, annexin-V-, and caspase-3-positive cells were determined. **c** Scatter plots showing the changes in the percentages of ethidium-III-, annexin-V-, or caspase-3-positive cells versus the changes in the mean nuclear γH2AX level (*circles* = compounds, *light red* = PTC-toxic compounds, *light blue* = non-PTC-toxic compounds, *error bars* = standard errors of the means, $\varDelta \mu$ = difference between the mean values of PTC-toxic and non-PTC-toxic compounds, *dashed lines* = optimum linear-regression fits of the data, *r* = Pearson’s correlation coefficient; all *P* values shown were obtained from one-sided *t* tests)

Is the DNA damage response associated with cell death under in vitro conditions? Based on the HPTC-B results, we selected three PTC-toxic compounds (cisplatin, cyclosporin A, and ochratoxin A) that induced increasing levels of γH2AX, and three non-PTC-toxic compounds (ribavirin, lithium chloride, and lincomycin) that caused small or no change in γH2AX levels (Fig. 4d). We treated primary PTCs with 1000 µg/mL of these compounds and labeled the cells with three different cell death markers: annexin-V [a marker for the externalization of phosphatidylserine, which occurs in both apoptotic and necrotic cells (Sawai and Domae 2011)], cleaved caspase-3 (a marker for the activation of caspase-3, which occurs only in apoptotic cells), and ethidium homodimer III (a DNA marker that is only permeant to late apoptotic or necrotic cells due to membrane disintegration; Fig. 5a). For each marker, we determined the percentages of positive cells under the treatments of these six compounds and solvent controls (Fig. 5b). Based on the HPTC-B dataset, we also determined the mean γH2AX nuclear levels of primary PTCs treated with 1000 µg/mL of these compounds. In agreement with our previous $\varDelta_{\hbox{max} }$ measurements, the three PTC-toxic compounds induced significantly higher mean γH2AX intensity levels than the three non-PTC-toxic compounds at the tested dosage (*P* = 0.044, Fig. 5c). However, only the increase in the percentage of annexin-V positive cells was significant (*P* = 0.047) between the PTC-toxic and non-PTC compounds. The increases in the percentages of ethidium homodimer III and caspase-3 positive cells were less significant (both *P* > 0.10, all one-sided *t* tests; Fig. 5c). This was mostly due to the lower cell death responses to cyclosporine A and ochratoxin A. Even for annexin-V, the responses were highly heterogeneous. For example, cyclosporine A and ochratoxin A only increased annexin-V levels in ~50 and ~25 % of the cells, respectively. These results corroborated with our earlier results on the heterogeneity in cyclosporine A responses (Fig. 4b, c). Surprisingly, the three PTC-toxic compounds only induced low percentages of caspase-3 positive cells (<20 %). Similar lack of apoptotic responses was also previously observed for some PTC-toxic compounds, such as 5-fluorouracil and gentamicin, in HK-2 cells (Wu et al. 2009). Across all the six compounds, we found that there is no significant positive correlation between γH2AX level and these three cell death markers (all *P* > 0.20, one-sided *t* test; Fig. 5c). Together, all of these results show that PTC toxicants induce variable cell death responses (both apoptosis and necrosis) under the tested in vitro conditions. Some of them (such as ochratoxin A, which induced a large increase in γH2AX levels) may even cause very small or no increase in cell death rates within the measured period. These results also imply that in vitro cell death endpoints may have difficulty in accurately predicting in vivo PTC toxicity and cannot be used to replace DNA damage features for nephrotoxicity prediction.

## Discussion

The current study shows that cell death of in vitro cultivated PTCs is induced to a variable degree by different PTC-toxic compounds (Fig. 5). This finding is in agreement with our and other previous results on predicting nephrotoxicity in humans (Wu et al. 2009; Li et al. 2013). The difficulties in using cell death as in vitro endpoint for predicting in vivo organ-specific toxicity may be related to the fact that in vivo compound-induced cell damage is not always associated with immediate cell death. For example, compound-induced PTC damage is often sublethal and associated with tubular dysfunction and chronic kidney disease instead of acute tubular necrosis (Kroshian et al. 1994; Choudhury and Ahmed 2006). The differences in the expression levels of xenobiotics-metabolizing enzymes and transporters may also play a role (Van der Hauwaert et al. 2014). Generally, it remains a challenge to identify highly predictive endpoints for in vitro organ-specific toxicity models (Lin and Will 2012). Specifically for kidney models, it has been consistently found that the use of general damage markers, such as ATP depletion; or potentially novel kidney-specific injury markers, such as kidney injury molecule-1 and neutrophil gelatinase-associated lipocalin, is of limited predictivity (Lin and Will 2012; Li et al. 2013; Tiong et al. 2014). The value of these current markers for predicting acute kidney injury remains to be controversially discussed (Bonventre et al. 2010; Vanmassenhove et al. 2013).

A commonly used strategy to address such difficulties is to achieve an improved understanding of organ-specific injury mechanisms and then select endpoints related to such mechanisms (Jennings et al. 2014). However, this requires a priori knowledge of injury mechanisms, which may not be known for novel or not well-characterized compounds. In the current study, we took a more pragmatic approach for nephrotoxicity prediction without requiring a priori characterization of injury mechanisms, and directly searched for in vitro phenotypic features that could best predict in vivo toxicity. The results were six sets of nuclear and actin cytoskeletal features that could achieve ~76–89 % test accuracies (Supplementary Material 1—Table S2). Our results show that, under in vitro conditions, most of the PTC-toxic compounds induce similar phenotypic changes in the nucleus and actin cytoskeleton, even though these compounds may have dissimilar chemical structures.

The identification of features associated with specific cellular changes provides a mechanistic interpretation for our computational models. One of the most surprising findings of our study is that γH2AX, which is a known marker for genotoxicity and carcinogenesis (Mah et al. 2010; Nikolova et al. 2014), was also induced by many compounds with diverse chemical structures. Some of our reference compounds, such as cisplatin, 5-fluorouracil and aristolochic acid, are known to directly interfere with DNA replication and cause double-strand breaks (Heidelberger et al. 1957; Lieberthal et al. 1996; Arlt 2002). However, most of our other reference PTC-toxic compounds are not known to interact with nucleic acids directly. Our observations are in agreement with other recent studies, which found that DNA damage responses were induced after renal ischemia–reperfusion injury in vivo and ATP depletion injury in vitro (Ma et al. 2014) and also after treatments of angiotensin II, which is not known to interact with DNA, in isolated perfused mouse kidneys and PTC cultures in vitro (Schmid et al. 2008). These observed DNA damage responses may be due to oxidative stress and/or oxidative DNA damage (Schmid et al. 2008; Ma et al. 2014). Some of our reference compounds, such as gentamicin, are known to induce oxidative stress and generate reactive-oxygen-species (ROS)-induced DNA damage (Quiros et al. 2011). Irrespective of the underlying molecular mechanisms, our study shows that in both primary PTCs and an immortalized PT cell line, γH2AX and DNA features were highly predictive of xenobiotics-induced PTC toxicity. Importantly, this also demonstrates how unexpected but common compound-induced cellular response and injury may be uncovered in an unbiased approach that does not focus on particular mechanisms.

Interestingly, the retainment of cytoskeleton features in our final feature sets suggests that the DNA/γH2AX and actin markers provide complimentary and non-redundant information about cellular responses to PTC-toxic compounds. Remodeling of the actin cytoskeleton induced by various types of toxic compounds has been reported in PTCs (Elliget et al. 1991; Kroshian et al. 1994). In addition to the cytoplasm, actin filaments are also localized in the nucleus, and actin-related proteins (Arps) are parts of chromatin remodeling complexes (Shen et al. 2003). Therefore, the possible role of actin filaments in DNA damage responses will be an important question for future research.

There were two main factors that contributed to the high accuracy of our computational models. The first factor was the use of image-based phenotypic features, which allowed us to quantitatively measure changes in the spatial organizations of cells, subcellular organelles, and biomolecules (such as DNA, histone modifications and actin cytoskeleton). We found that Haralick’s texture features of the chromatin and cytoskeleton contained highly discriminative information, which would be lost under population-averaged or non-image-based measurements. Our results also show that the initial set of 129 general phenotypic features was a good starting point for screening predictive toxicity endpoints. The second factor that contributed to the high accuracy was the design of our reference compounds and performance evaluation methodology (Methods). The inclusion of diverse compounds and non-PTC-toxic toxicants in the negative reference groups allowed us to search for more specific phenotypic features. We also ensured that training and test data were statistically independent from each other. For example, the feature normalization and elimination parameters were always determined using the training data only, but applied to both the training and test data in every single fold in our cross-validation procedure.

Our nephrotoxicity models may be further improved by the following ways. First, bioactivations of many xenobiotics are required for their in vivo toxicity effects (Van der Hauwaert et al. 2014). Better culturing methods or conditions may improve the ability of PTCs to transport and metabolize these compounds under in vitro conditions (Leite et al. 2012). Second, the test accuracies of our models were often lower than their training accuracies, suggesting our models may be slightly overfitted and the estimated performances of our models may be conservative. Increasing the number of reference compounds or using more conservative feature selection criteria (Fig. 3e) may reduce the overfitting. Lastly, we used recursive feature elimination, which is a heuristic algorithm, for feature selection. More complex algorithms, such as genetic algorithms (Siedlecki and Sklansky 1989) or floating search methods (Pudil et al. 1994) may identify more predictive feature subsets, but at the expense of much higher computational cost. In conclusion, our study demonstrates the feasibility of predicting the human nephrotoxicity of xenobiotic compounds with diverse chemical structures using high-throughput imaging, phenotypic profiling, and machine learning methods.

## Materials and methods

### Reference compounds

For the HPTC-A dataset (DNA/RelA/actin/WCS), we used 44 xenobiotic compounds. The “PTC-toxic” group had 24 nephrotoxicants known to damage human proximal tubular cells (PTCs) in vivo, and the “non-PTC-toxic” group had 12 nephrotoxicants not known to damage PTCs and 8 non-nephrotoxicants [detailed information on the PTC toxicity of most of the compounds can be found in our reports (Li et al. 2014; Kandasamy et al. 2015)]. For the HPTC-B and HK-2 datasets (DNA/γH2AX/actin/WCS), 42 of the compounds were used (excluding lead acetate and hydrocortisone). The compounds were dissolved in either DMSO at a stock concentration of 50 mg/mL, or water at a stock concentration of 10 mg/mL. The full list of reference compounds and their sources, solvents, and known human kidney and liver toxicity are provided in Supplementary Material 1—Table S1.

### Cell culture and compound treatment

For both the HPTC-A and HPTC-B datasets, we used three different batches of primary human PTCs from three different donors. Two of them (HPTC1 and HPTC10; Lot #58488852 and #61247356, respectively) were bought from the American Type Culture Collection (ATCC, Manassas, VA, USA). The third batch of cells (HPTC6) was isolated from a human nephrectomy sample (National University Health System, Singapore). Only normal tissues without aberrant pathological changes, as determined by a pathologist, were used. Ethics approvals for the work with primary human kidney samples (DSRB-E/11/143) and cells (NUS-IRB Ref. Code: 09-148E) were obtained. All three batches of primary PTCs were cultured in renal epithelial cell basal medium (ATCC) supplemented with renal epithelial cell growth kit (ATCC) and 1 % penicillin/streptomycin (Gibco, Carlsbad, CA, USA). Only passages (P) 4 and P5 of primary PTCs were used in this study. For the HK-2 dataset, the HK-2 cell line (ATCC) was maintained in Dulbecco’s modified eagle medium (DMEM) supplemented with 10 % fetal bovine serum (FBS; Gibco) and 1 % penicillin/streptomycin.

Cells were seeded into 384-well black plates with transparent bottom (Greiner, Kremsmünster, Austria). All cells were cultured for 3 days to achieve the formation of a differentiated renal epithelium before overnight drug treatment (16 h; Li et al. 2013). The dosages of the tested compounds were 1.6, 16, 63, 125, 250, 500, 1000 μg/mL. Positive, negative, and vehicle controls (DMSO or water, depending on the solvent of the tested compounds) and untreated cells were included on each plate. Four technical replicates were performed for each compound and dosage.

### Immunostaining

After compound treatment for 16 h, cells were fixed using 3.7 % formaldehyde in phosphate-buffered saline (PBS). The cells were blocked for 1 h with PBS containing 5 % bovine serum albumin (BSA) and 0.2 % Triton X-100. The samples were incubated with a mouse monoclonal antibody to γH2AX (phospho S139) (Abcam, Cambridge, MA, USA) at 2 µg/mL, or a rabbit polyclonal antibody to RelA (Abcam) at 1 µg/mL for 1 h at room temperature. Subsequently, the cells were incubated with a goat anti-mouse secondary antibody conjugated to Alexa 488 (Abcam) or a goat anti-rabbit secondary antibody conjugated to Alexa488 (Life Technologies, Carlsbad, CA, USA) at 5 µg/mL. Finally, the cells were stained with DAPI (Merck Millipore, Darmstadt, Germany) at 4 µg/mL, rhodamine phalloidin (Life Technologies) and whole-cell stain red (Life Technologies).

### Apoptosis and necrosis assays

Cells were seeded into 96-well black plates with transparent bottom (Falcon, Corning, NY, USA) and cultured for 3 days before overnight drug treatment (16 h). They were treated with cisplatin, cyclosporin A, ochratoxin A, lincomycin, lithium chloride and ribavirin at 1000 μg/mL. Untreated cells and vehicle controls (DMSO and water) were included on each plate as well as positive (25 μg/mL arsenic(III) oxide) and negative (100 μg/mL dexamethasone) controls. Three technical replicates were performed for each treatment condition.

Cleaved caspase-3 (Abcam) and apoptotic/necrotic/healthy cells detection kits (PromoKine, Heidelberg, Germany) were used to identify apoptotic and necrotic cells. For cleaved caspase-3, the same immunostaining protocol as outlined above was used. The rabbit polyclonal anti-cleaved-caspase-3 antibody was diluted in blocking buffer and incubated with fixed cells for 1 h in room temperature. The cells were then incubated with a goat anti-rabbit secondary antibody conjugated to Alexa 488 at 5 µg/mL. Finally, the cells were counterstained with DAPI at 4 µg/mL and whole-cell stain red. For the apoptotic/necrotic/healthy cells detection kit, the protocols provided by manufacturer were used.

### Image acquisition

Imaging was performed with a 20 × objective using the ImageXpress Micro XLS system (Molecular Devices, Sunnyvale, CA, USA). Four different channels were used to image DAPI, Alexa 488, Texas Red, and Cy5 fluorescence. Nine sites per well were imaged. The images were saved in 16-bit TIFF format.

### Image segmentation and feature extraction

To reduce non-uniform background illuminations, we corrected the images using the “rolling ball” algorithm implemented in ImageJ (NIH, v1.48; Sternberg 1983). Cell segmentations and feature measurements were performed using the cellXpress software platform (Bioinformatics Institute, v1.2; Laksameethanasan et al. 2013). We extracted 129 features, which include 78 Haralick texture features, 29 intensity features, 9 intensity ratio features, 6 correlation features, 6 morphology features and cell count from the images. The detail list of features and their markers is shown in Supplementary Material 2.

### Haralick’s texture features

The mathematical definitions of all Haralick’s texture features were described in Haralick’s original paper (Haralick et al. 1973). Here, we only provide mathematical definitions for the Haralick’s features included in our final feature sets. A gray-level co-occurrence matrix (GLCM) is a matrix that describes the distribution of co-occurring gray-level values at a given offset $(\varDelta x,\varDelta y)$ in an $N_{x} \times N_{y}$ image, ${\mathbf{I}}(x,y)$, with $N_{g}$ gray levels. In our notations, $x$ and $y$ are the row and column indices, respectively. The GLCM matrix is defined by

$${\mathbf{GLCM}}_{\varDelta x,\varDelta y} (i,j) = \sum\limits_{x = 1}^{{N_{x} }} {\sum\limits_{y = 1}^{{N_{y} }} {\left\{ {\begin{array}{ll} {1\;,} & {{\text{if }}{\mathbf{I}}(x,y) = i\;\;{\text{and}}\;\;{\mathbf{I}}(x + \varDelta x,\,y + \varDelta y) = j} \\ {0\;,} & {\text{otherwise}} \\ \end{array} } \right.} } ,$$

where $i$ and $j$ are the gray-level or intensity values of the image. The normalized GLCM matrix is

$$p(i,j,\varDelta x,\varDelta y) = \frac{{{\mathbf{GLCM}}_{\varDelta x,\varDelta y} (i,j)}}{{\sum\nolimits_{i = 1}^{{N_{g} }} {\sum\nolimits_{j = 1}^{{N_{g} }} {{\mathbf{GLCM}}_{\varDelta x,\varDelta y} (i,j)} } }}$$

Then, we have the marginal and sum probability matrices to be $p_{x} (j,\varDelta x,\varDelta y) = \sum\nolimits_{i = 1}^{{N_{g} }} {p(i,\,j,\,\varDelta x,\,\varDelta y)}$, $p_{y} (i,\varDelta x,\varDelta y) = \sum\nolimits_{j = 1}^{{N_{g} }} {p(i,\,j,\,\varDelta x,\,\varDelta y)}$, and $p_{x + y} (k,\varDelta x,\varDelta y) = \mathop {\sum\nolimits_{i = 1}^{{N_{g} }} {\sum\nolimits_{j = 1}^{{N_{g} }} {} } }\nolimits_{i + j = k} p(i,\,j,\varDelta x,\varDelta y),$ where $\,k = 2,\,3,\, \ldots ,\,2N_{g} .$

The Haralick’s features are

- Angular second moment: $f_{\text{ASM}} (\varDelta x,\varDelta y) = \sum\nolimits_{i} {\sum\nolimits_{j} {\left\{ {p(i,j,\varDelta x,\varDelta y)} \right\}^{2} } }$
- Correlation: $f_{\text{COR}} (\varDelta x,\varDelta y) = \frac{1}{{\sigma_{x} \sigma_{y} }}\sum\nolimits_{i} {\sum\nolimits_{j} {(i\,j)p(i,j,\varDelta x,\varDelta y)} } - \mu_{x} \mu_{y}$, where $\mu_{x}$, $\mu_{y}$, $\sigma_{x}$ and $\sigma_{y}$ are the means and standard deviations of $p_{x} (j,\varDelta x,\varDelta y)$ and $p_{y} (i,\,\varDelta x,\varDelta y)$, respectively.
- Sum average: $$f_{\text{SA}} (\varDelta x,\varDelta y) = \sum\nolimits_{k = 2}^{{2N_{g} }} {k\,p_{x + y} (k,\varDelta x,\varDelta y)}$$
- Sum variance: $$f_{\text{SV}} (\varDelta x,\varDelta y) = \sum\nolimits_{k = 2}^{{2N_{g} }} {(k - f_{\rm SA} (\varDelta x,\varDelta y))^{2} \,p_{x + y} (k,\varDelta x,\varDelta y)}$$
- Sum entropy: $$f_{\text{SE}} (\varDelta x,\varDelta y) = - \sum\nolimits_{k = 2}^{{N_{g} }} {p_{x + y} (k,\varDelta x,\varDelta y)\;\log \left[ {p_{x + y} (k,\varDelta x,\varDelta y)} \right]}$$
- Entropy: $$f_{E} (\varDelta x,\varDelta y) = - \sum\nolimits_{i} {\sum\nolimits_{j} {p(i,j,\varDelta x,\varDelta y)\;} } \log [p(i,j,\varDelta x,\varDelta y)]$$
- Information measure of correlation 2: $f_{{{\text{IMC}}2}} (\varDelta x,\varDelta y) = \sqrt {\left| {1 - \exp \left[ { - 2\left( {{\text{HXY}}2 - f_{E} (\varDelta x,\varDelta y)} \right)} \right]} \right|}$, where ${\text{HXY}}2 = - \sum\nolimits_{i} {\sum\nolimits_{j} {p_{x} (j,\varDelta x,\varDelta y)p_{y} (i,\varDelta x,\varDelta y)\log \left[ {p_{x} (j,\varDelta x,\varDelta y)p_{y} (i,\varDelta x,\varDelta y)} \right]} }$

In our study, the images were the bounding boxes around the segmented cells with all the background pixels set to zero. We quantized the images into $N_{g} = 256$ gray levels, and computed all the Haralick’s features for 0° $\left( {\varDelta x = 0,\varDelta y = 1} \right)$, 45° $\left( {\varDelta x = 1,\varDelta y = 1} \right)$, 90° $\left( {\varDelta x = 1,\varDelta y = 0} \right)$, and 135° $\left( {\varDelta x = 1,\varDelta y = - 1} \right)$ offsets. For each feature, the mean and standard deviation of the feature across all the offset values were used. We have implemented the extraction procedures for all the features using C++ in the cellXpress software platform (Bioinformatics Institute, v1.2; Laksameethanasan et al. 2013).

### Concentration response curve and Δ_max_ estimations

After feature extraction, we divided the values of a feature at all the tested compound concentrations by the values of the feature under the corresponding vehicle control conditions. Then, the ratios were log 2-transformed (Δ). All further data analyses, including building concentration response curves and toxicity classifiers, were performed using customized scripts under the R statistical environment (the R foundation, v3.0.2) and the Windows 7 operating system (Microsoft, USA).

For each feature, we estimated its concentration response curve using a standard log-logistic model:

$$\varDelta (x,(b,c,d,e)) = \frac{d - c}{{1 + \exp \{ b(\log (x) - \log (e))\} }},$$

where *x* is the xenobiotics compound concentration, *e* is the response half-way between the lower limit *c* and upper limit *d*, and *b* is the relative slope around *e*. We used the “drc” library (v 2.3-96) under the R environment to fit the values of *b*, c, *d*, and *e*. After that, the maximum response values ($\varDelta_{\hbox{max} }$) were determined using the estimated response curves. In theory, $\varDelta_{\hbox{max} }$ should be equal to the upper limit *d*. However, in practice, the responses of some compounds may not plateau even at the highest tested dosages, and therefore the estimated *d* value may not be accurate. Instead, we fixed $\varDelta_{\hbox{max} }$ to be the response value at 5 mM, which was around the highest tested concentrations for most of the our compounds. Finally, the median values of $\varDelta_{\hbox{max} }$ across the three biological replicates were computed. The final result was a 129 × 44 (or 42) matrix of $\varDelta_{\hbox{max} }$ values, which were used for training and testing the classifiers. Each column of the matrix was a feature vector, ${\mathbf{f}}_{i}$, where $i = 1,\,2,\, \ldots ,\,129$.

### Feature normalization

Before data classification, each feature vector ${\mathbf{f}}_{i}$ was normalized to the same range [−1, 1]:

$${\mathbf{f}}_{i} \leftarrow 2\frac{{({\mathbf{f}}_{i} - f_{\hbox{min} } )}}{{f_{\hbox{max} } - f_{\hbox{min} } }} - 1,$$

where $f_{\hbox{min} }$ and $f_{\hbox{max} }$ are the minimum and maximum values of the feature. To ensure the training and test datasets were independent to each other, these two normalization coefficients were estimated only using the training data, but applied to both training and test datasets.

### Random forest classification

We used the random-forest algorithm (Breiman 2001) to predict xenobiotic-induced nephrotoxicity, because we have previously shown that the algorithm outperforms other commonly used classifiers, including support vector machine, *k*-nearest neighbors and naïve Bayes (Su et al. 2014). A random forest has two main parameters: $N_{\text{tree}}$ and $N_{\text{trial}}$. The first parameter specifies the number of decision trees built, and the second parameter specifies the number of random features used at each level of the decision trees. During cross-validation, we automatically determine the optimum classifier parameters using a grid search for $N_{\text{tree}} = \left\{ {10,50,150,250,400,500} \right\}$ and $N_{\text{trial}} = \left\{ {1,2,3,4,5} \right\}$. A series of temporary random forests were trained using all the possible combinations of parameters based on a training dataset $\bar{\mathbf{X}}^{\prime}_{\text{training}}$, and the test accuracies of these combinations were estimated based on an independent test dataset $\bar{\mathbf{X}}^{\prime}_{\text{FStest}}$. The combination of $N_{\text{tree}}$ and $N_{\text{trial}}$ with the highest test accuracy value were selected to train a final classifier, whose performance would then be estimated using a third independent test dataset $\bar{\mathbf{X}}^{\prime}_{\text{RFtest}}$. We used the “randomForest” library (v4.6-10) under the R environment.

### Automated feature selection

We used a greedy search algorithm, namely recursive feature elimination (RFE; Loo et al. 2007), to select a subset of features from all the extracted features ${\mathbf{F}}_{\text{all}} = \{ {\mathbf{f}}_{1} ,{\mathbf{f}}_{2} , \ldots ,{\mathbf{f}}_{{m_{\text{all}} }} \}$. The pseudocode for the algorithm is listed in Algorithm S2 (Supplementary Material 1—Text S1). The main idea is to start with all the features, iteratively rank the current feature set, remove the least important feature subset, evaluate the accuracy ${\text{acc}}_{j}$ of the retained feature subset ${\mathbf{F}}_{j}$ and finally select the feature subset with the highest accuracy. To reduce data overfitting, the ranking and evaluation of feature subsets were performed in two independent datasets, $\left\{ \bar{\mathbf{X}}^{\prime}_{\text{training}} ,\,\bar{\mathbf{X}}^{\prime}_{\text{FStest} } \right\}$ and $\bar{\mathbf{X}}^{\prime}_{\text{RFtest}}$, respectively (Algorithm S2). We ranked features based on their importance values estimated by the random forest algorithm by permuting the out-of-bag data and features (Breiman 2001).

In datasets with small sample sizes, the ${\text{acc}}_{j}$ curve (as a function of ${\mathbf{F}}_{j}$) may not be smooth. Thus, the global maxima of ${\text{acc}}_{j}$ may not be a robust criterion for selecting the final feature subset. Instead, we designed an automated procedure to select a feature subset using Gaussian mixture modeling (GMM; Trevor Hastie et al. 2009). We clustered all the ${\text{acc}}_{j}$ values into 2–4 groups. Each of them was modeled as a Gaussian distribution. Then, we selected the smallest feature subset in the group with the highest average prediction accuracy (Algorithm S2). The optimum number of groups was also automatically determined based on the Bayesian information criterion (BIC), ${\text{BIC}} = - 2L_{m} + N_{d} \log \,(N_{s} )$, where $N_{s}$ is the sample size, *L*_*m*_ is the maximum log-likelihood computed by the GMM algorithm, and $N_{d}$ is the number of the parameters.

### Classification performance estimation

We used a stratified tenfold cross-validation procedure (Trevor Hastie et al. 2009) to estimate the PTC toxicity prediction performance of our phenotypic features. The pseudocode for the procedure is listed in Algorithm S1 (Supplementary Material 1—Text S1). The procedure has two main cross-validation loops. The first cross-validation loop aims to identify an optimum feature subset ${\mathbf{F}}_{\text{final}}$, while the second cross-validation loop aims to estimate the generalized prediction performance of ${\mathbf{F}}_{\text{final}}$. To keep the training and test data independent from each other, we divided all the treatment conditions into four non-overlapping sets, ${\mathbf{X}}_{\text{training}} ({\mathbf{F}}_{\text{all}} )$, ${\mathbf{X}}_{\text{FStest}} ({\mathbf{F}}_{\text{all}} )$, ${\mathbf{X}}_{\text{RFtest}} ({\mathbf{F}}_{\text{all}} )$, and ${\mathbf{X}}_{\text{test}} ({\mathbf{F}}_{\text{all}} )$. Furthermore, the normalization coefficients and classifier parameters were always estimated based on the training datasets only, but applied to both training and test datasets.

We used the following performance measurements

$$\begin{aligned} {\text{Sensitivity}} & = \frac{\text{TP}}{{{\text{TP}} + {\text{FN}}}} \times 100\;\% , \\ {\text{Specificity}} & = \frac{\text{TN}}{{{\text{TN}} + {\text{FP}}}} \times 100\;\% ,\quad {\text{and}} \\ {\text{Balanced}}\;{\text{accuracy}}\;({\text{acc}}) & = \frac{{{\text{Sensitivity}} + {\text{Specificity}}}}{2}, \\ \end{aligned}$$

where TP is the number of true positives, TN is the number of true negatives, FP is the number of false positives and FN is the number of false negatives. The same performance estimation procedure was used for HPTC-A, HPTC-B and HK-2 datasets.

### Multi-dimensional scaling plots

To compare the compounds in the chemical structure space, we used the ChemmieR library to compute the pairwise Tanimoto coefficients between the structures of all the reference compounds. To compare the compounds in the phenotypic feature space, we first scaled all the phenotypic features to the same range [0, 1] and then computed the pairwise Euclidean distances between the feature values of all the reference compounds. Finally, we used the cmdscale function (Torgerson 1952) in the R environment to generate the multi-dimensional scaling plots.

## Electronic supplementary material

Below is the link to the electronic supplementary material.

Supplementary material 1 (PDF 1387 kb)

Supplementary material 2 (XLS 109 kb)

Supplementary material 3 (XLS 340 kb)
