# Supplementary material for: High-throughput imaging-based nephrotoxicity prediction for xenobiotics with diverse chemical structures
Source: Arch Toxicol. 2015 Nov 27;90(11):2793–808. doi: 10.1007/s00204-015-1638-y (PMC5065616; doi:10.1007/s00204-015-1638-y)
Supplement: Supplementary file 1 — Supplementary material 1 (PDF 1387 kb) [file 204_2015_1638_MOESM1_ESM.pdf]

Figure S1. Overview of our image and data analysis procedures

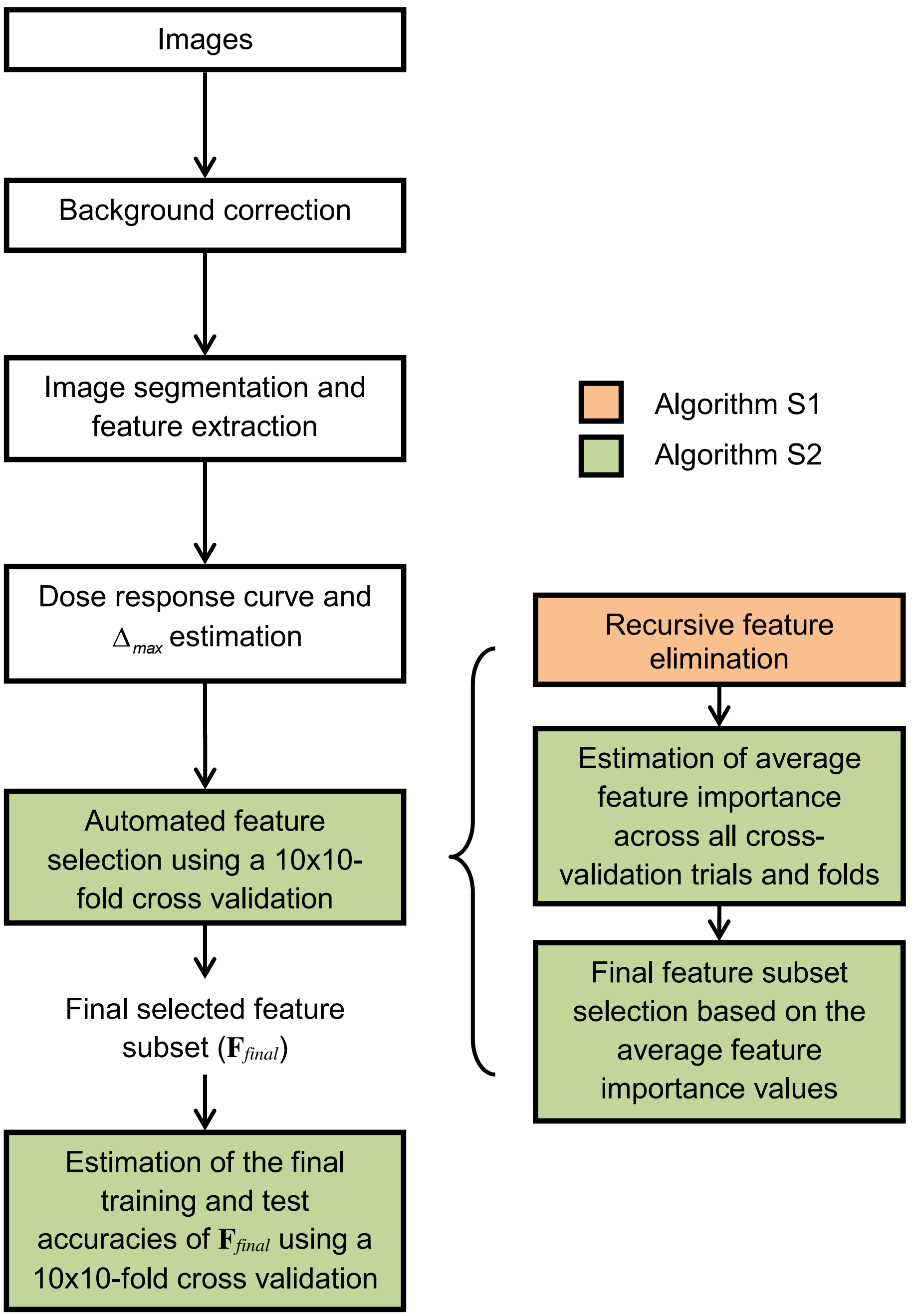

Figure S2. Automated cell segmentation

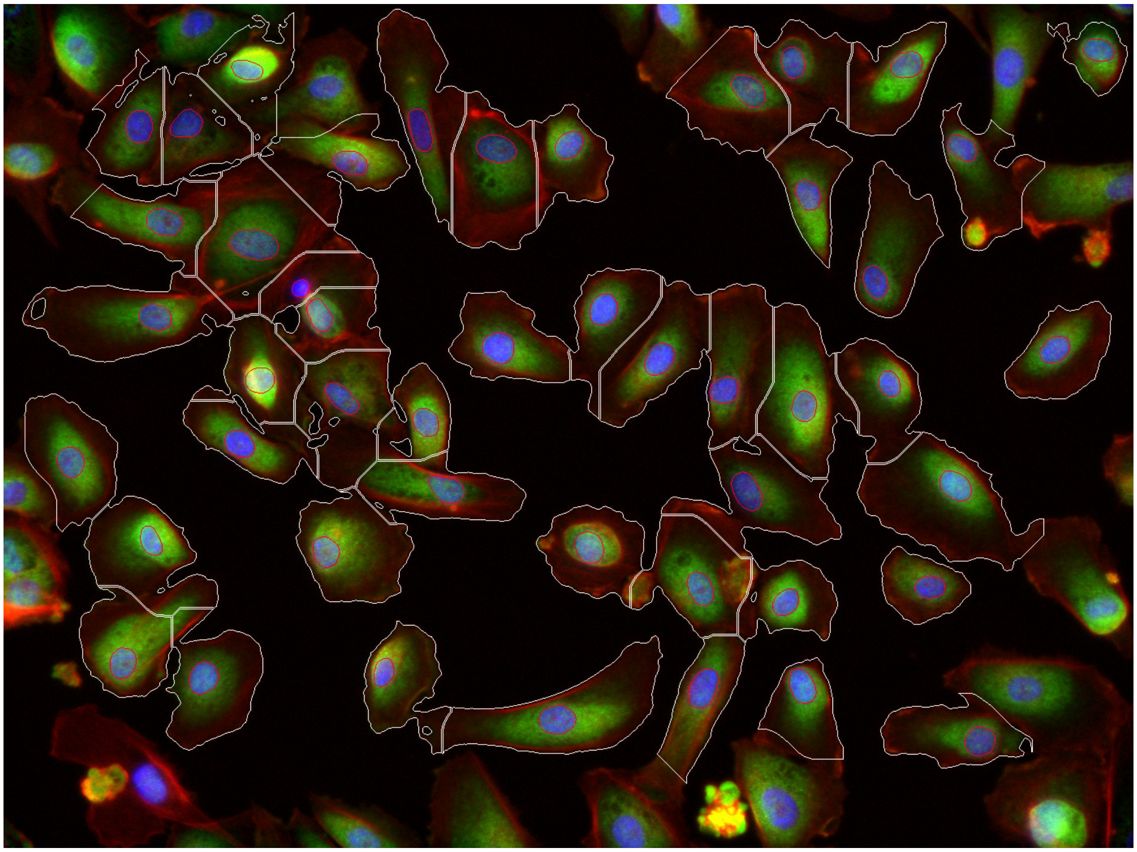

An example of a full-frame immunofluorescence image showing automatically identified cell boundaries (white lines) and nuclear boundaries (red lines) of primary human proximal tubule cells (blue = DNA marker, green = RelA marker, red = actin marker, the whole-cell stain marker is not shown.) Cells that touched the image boundary were not included in our analysis.

Figure S3. Training versus test accuracies of single-feature classifiers

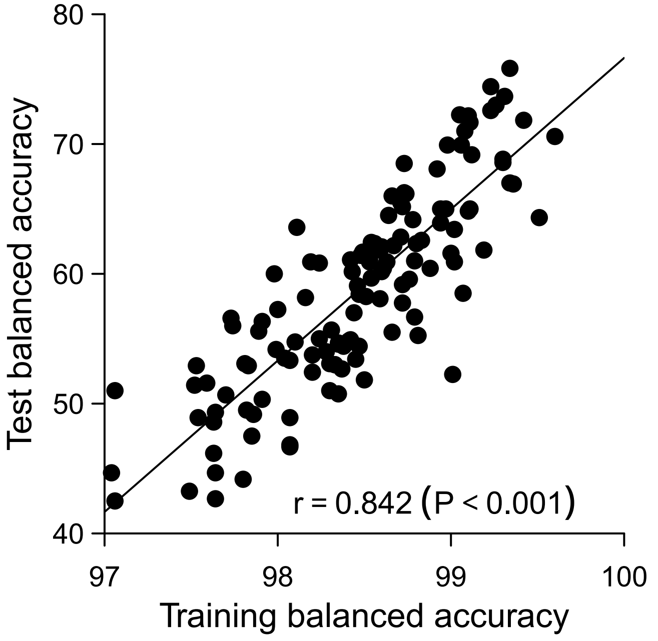

The training and test accuracies of all the single-feature classifiers for the HPTC-A dataset (line = optimum linear-regression fit of the data, r = Pearson's correlation coefficient).

Figure S4. Spatial distribution patterns represented by the best single features

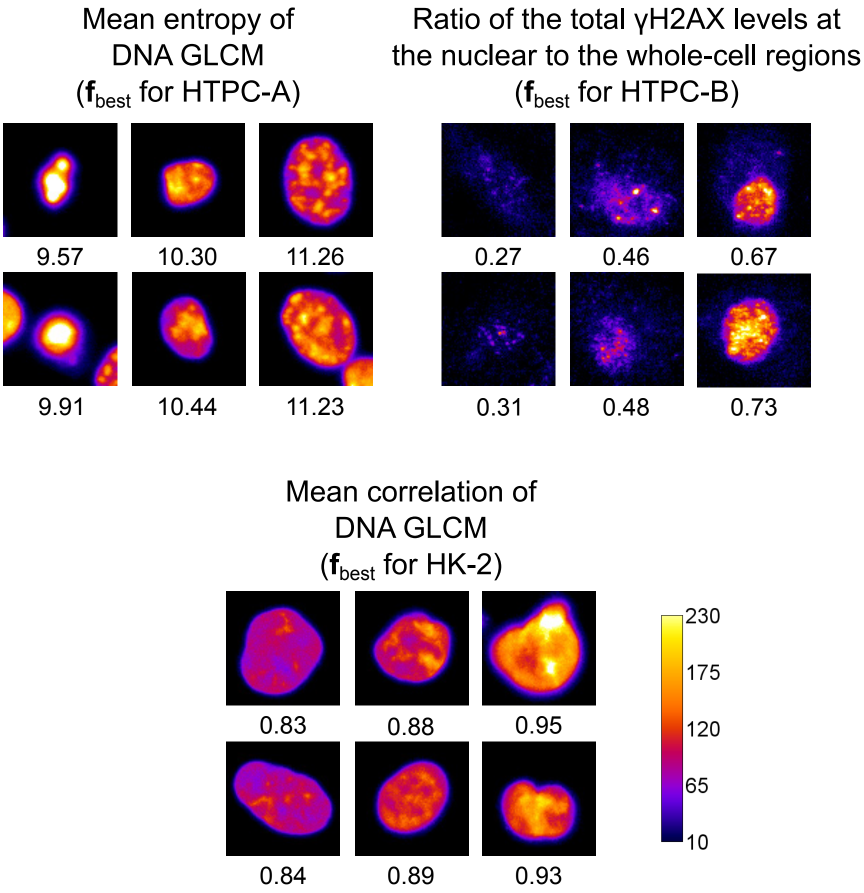

Exemplary immunofluorescence images showing the spatial distribution patterns represented by the best single features ($\mathbf{f}_{best}$) for all three datasets. Cells with varying (left=low, center=middle, and right=high) values of the features were shown. The feature values quantified from the images are shown below the respective images.

Figure S5. Average importance values of the final selected features

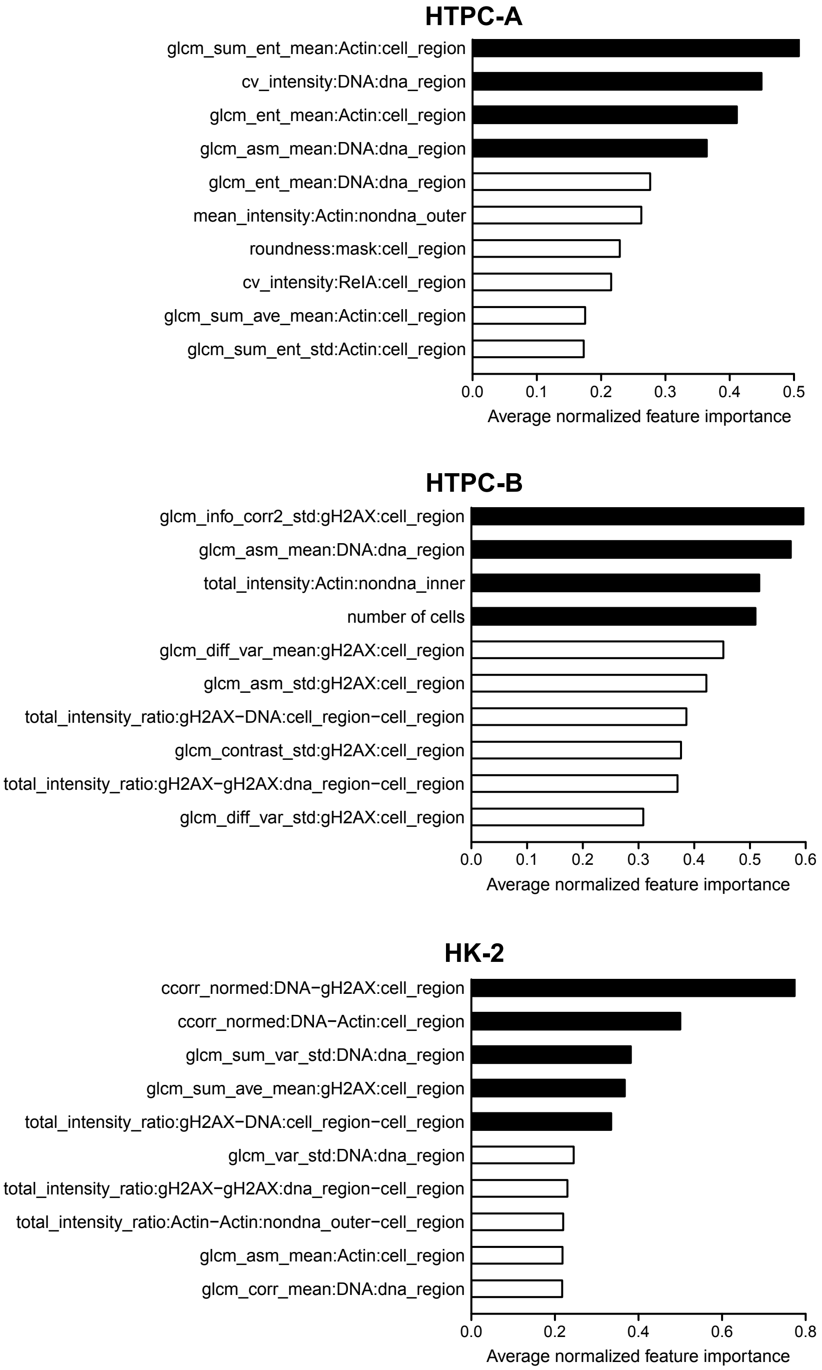

Average feature importance values estimated using a 10x10 cross validation procedure (**Algorithm S1**) for the three datasets. Only the top ten features are shown (black bars = final selected features (**F**$_{final}$), white bars = other top ten features.) The feature names are shown in the cellXpress format, and a detailed description of the final selected features is included in the footnote of **Table S2**.

Figure S6. γH2AX and DNA staining patterns in human HK-2 cells treated with PTC-toxic or non-PTC-toxic compounds

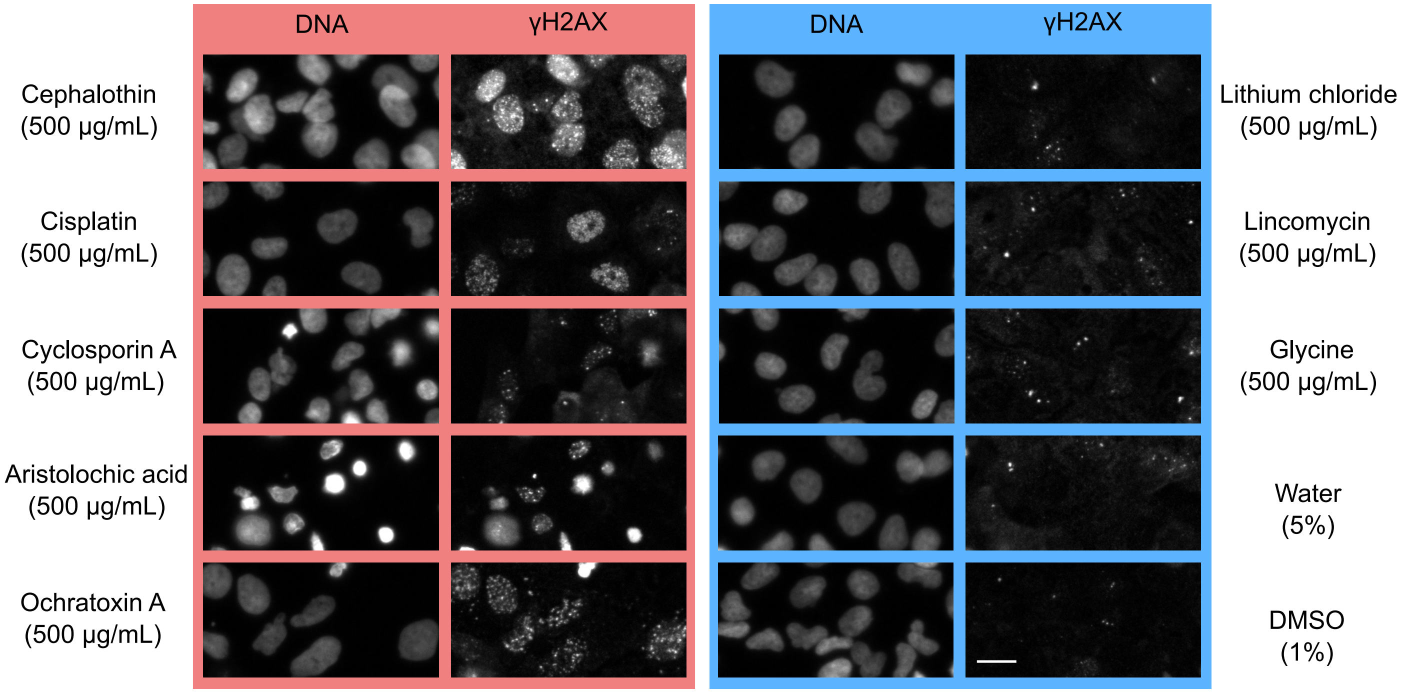

Immunofluorescence microscopy images showing the γH2AX and DNA staining patterns in human HK-2 cells treated with five PTC-toxic compounds (light red), three non-PTC-toxic compounds (blue), and two solvent controls (light blue). All images from the same markers have the same exposure times and display intensity ranges (scale bar = 20 μm).

Table S1. Reference compound information

Drug name	CAS number	PTC-toxic	Nephrotoxic	Hepatotoxic	Category	HPTC-A	HPTC-B/HK2	Source	Catalog no.	Solvent *
5-Fluorouracil	51-21-8	1	1	1	Chemotherapy drugs	Y	Y	Sigma-Aldrich	F6627	DMSO
Acarbose	56180-94-0	0	0	1	Anti-diabetic drugs	Y	Y	Sigma-Aldrich	A8980	water
Acetaminophen	103-90-2	0	1	1	Anti-inflammatory drugs	Y	Y	Sigma-Aldrich	A3035	water
Aristolochic acid	313-67-7	1	1	1	Herbs	Y	N	Calbiochem	182300	DMSO
Arsenic(III) oxide	1327-53-3	1	1	1	Industrial chemicals	Y	Y	Sigma-Aldrich	202673	water
Bismuth(III) oxide	1304-76-3	1	1	1	Industrial chemicals	Y	Y	Sigma-Aldrich	637017	water
Cadmium(II) chloride	10108-64-2	1	1	1	Industrial chemicals	Y	Y	Sigma-Aldrich	202908	water
Cephaloridine	50-59-9	1	1	0	Antibiotics	Y	Y	Creative Dynamics	50-59-9	water
Cephalosporin C	61-24-5	1	1	0	Antibiotics	Y	Y	Sigma-Aldrich	C3270	water
Cephalothin	153-61-7	1	1	0	Antibiotics	Y	Y	Sigma-Aldrich	C4520	water
Ciprofloxacin	85721-33-1	0	1	1	Antibiotics	Y	Y	Sigma-Aldrich	17850	water
Cisplatin	15663-27-1	1	1	0	Chemotherapy drugs	Y	Y	Calbiochem	232120	DMSO
Citrinin	518-75-2	1	1	0	Mycotoxins	Y	Y	Sigma-Aldrich	C1017	DMSO
Copper(II) chloride	7447-39-4	1	1	1	Industrial chemicals	Y	Y	Merck Millipore	818247	water
Cyclosporin A	59865-13-3	1	1	1	Immunosuppressants	Y	Y	Calbiochem	239835	DMSO
Dexamethasone	50-02-2	0	0	0	Steroids	Y	Y	Sigma-Aldrich	D2915	water
Ethylene glycol	107-21-1	0	1	0	Industrial chemicals	Y	Y	EMSURE	109621	water
Furosemide	54-31-9	0	1	1	Other drugs	Y	Y	Sigma-Aldrich	F4381	DMSO
Gentamicin	1403-66-3	1	1	0	Antibiotics	Y	Y	PAA	P11-004	water
Germanium(IV) oxide	1310-53-8	1	1	0	Industrial chemicals	Y	Y	Sigma-Aldrich	199478	water
Glycine	56-40-6	0	0	0	Food additives	Y	Y	Sigma-Aldrich	410225	water
Gold(I) chloride	10294-29-8	1	1	1	Industrial chemicals	Y	Y	Sigma-Aldrich	481130	water
Hydrocortisone	50-23-7	0	0	0	Steroids	Y	Y	Sigma-Aldrich	H0888	DMSO
Ibuprofen	15687-27-1	0	1	1	Anti-inflammatory drugs	Y	Y	Sigma-Aldrich	14883	DMSO
Lead(IV) acetate	546-67-8	1	1	1	Industrial chemicals	Y	Y	Sigma-Aldrich	185191	DMSO
Levodopa	59-92-7	0	0	0	Psychoactive drugs	Y	Y	Sigma-Aldrich	D9628	water
Lincomycin	154-21-2	0	1	0	Antibiotics	Y	Y	Sigma-Aldrich	62143	water
Lindane	58-89-9	0	1	0	Agricultural chemicals	Y	Y	Sigma-Aldrich	49049	DMSO
Lithium chloride	7447-41-8	0	1	0	Industrial chemicals	Y	Y	Calbiochem	438002	water
Melatonin	73-31-4	0	0	0	Psychoactive drugs	Y	Y	Calbiochem	444300	DMSO
Metformin hydrochloride	657-24-9	0	1	0	Anti-diabetic drugs	Y	Y	Sigma-Aldrich	1396309	water
Ochratoxin A	303-47-9	1	1	0	Mycotoxins	Y	Y	Sigma-Aldrich	O1877	DMSO
Paraquat	1910-42-5	1	1	1	Agricultural chemicals	Y	Y	Sigma-Aldrich	PS-366	water
Phenacetin	62-44-2	0	1	1	Anti-inflammatory drugs	Y	Y	Sigma-Aldrich	77440	DMSO
Potassium dichromate	7778-50-9	1	1	1	Industrial chemicals	Y	Y	EMSURE	1.04864.0500	water
Puromycin	53-79-2	1	1	1	Antibiotics	Y	Y	Calbiochem	540411	water
Ribavirin	36791-04-5	0	0	0	Antivirals	Y	Y	Calbiochem	555580	water
Rifampicin	13292-46-1	1	1	1	Antibiotics	Y	Y	Calbiochem	557303	DMSO
Tacrolimus	104987-11-3	1	1	0	Immunosuppressants	Y	Y	Calbiochem	342500	DMSO
Tenofovir	147127-20-6	1	1	0	Antivirals	Y	N	Tocris Bioscience	3666-50	water
Tetracycline	60-54-8	1	1	1	Antibiotics	Y	Y	Calbiochem	583411	water
Triiodothyronine	6893-02-3	0	0	0	Psychoactive drugs	Y	Y	Calbiochem	642511	DMSO
Valacyclovir	124832-26-4	0	1	1	Antivirals	Y	Y	Sigma-Aldrich	1707839	water
Vancomycin	1404-90-6	0	1	1	Antibiotics	Y	Y	Calbiochem	627850	water

* DMSO = 50mg/mL in DMSO, Water = 10mg/mL in water

Table S2. Summary of the overall prediction performances for single- and multi-feature classifiers for all three datasets

Dataset	Markers	Compound number	Feature number	Feature names (cellXpress format)	Balanced accuracy		Sensitivity		Specificity	
					Training	Test	Training	Test	Training	Test
HPTC-A	DNA/RelA/Actin/WCS	44	1	glcm_ent_mean:DNA:dna_region	99.3	75.8	99.6	81.2	99.1	70.5
HPTC-A	DNA/RelA/Actin/WCS	44	4	glcm_sum_ent_mean:Actin:cell_region, cv_intensity:DNA:dna_region, glcm_ent_mean:Actin:cell_region, glcm_asm_mean:DNA:dna_region	99.5	78.3	99.4	76.5	99.7	80.0
HPTC-A	DNA/RelA/Actin/WCS	42	4	glcm_sum_ent_mean:Actin:cell_region, cv_intensity:DNA:dna_region, glcm_ent_mean:Actin:cell_region, glcm_asm_mean:DNA:dna_region	99.6	77.8	99.7	75.2	99.6	80.5
HPTC-B	DNA/γH2AX/Actin/WCS	42	1	total_intensity_ratio:gH2AX-gH2AX:dna_region-cell_region	99.5	77.6	99.4	72.2	99.7	83.0
HPTC-B	DNA/γH2AX/Actin/WCS	42	4	total_intensity:Actin:nondna_inner, glcm_asm_mean:DNA:dna_region, glcm_info_corr2_std:gH2AX:cell_region, cellcount	99.7	81.6	99.9	83.7	99.6	79.5
HK-2	DNA/γH2AX/Actin/WCS	42	1	glcm_corr_mean:DNA:dna_region	99.8	83.9	99.6	78.3	99.9	89.5
HK-2	DNA/γH2AX/Actin/WCS	42	5	ccorr_normed:DNA-gH2AX:cell_region, ccorr_normed:DNA-Actin:cell_region, glcm_sum_ave_mean:gH2AX:cell_region , total_intensity_ratio:gH2AX-DNA:cell_region-cell_region, glcm_sum_var_std:DNA:dna_region	99.9	88.9	100.0	98.8	99.8	79.0

Note:

Feature names (cellXpress format)	Descriptions
ccorr_normed:DNA-gH2AX:cell_region	Normalized spatial correlation coefficient between DNA and γH2AX intensities at the whole-cell region
ccorr_normed:DNA-Actin:cell_region	Normalized spatial correlation coefficient between DNA and actin intensities at the whole-cell region
cellcount	Cell count
cv_intensity:DNA:dna_region	Coefficient of variation (CV) of the DNA intensity at the nuclear region
glcm_asm_mean:DNA:dna_region	Mean angular second moment (ASM) of DNA GLCM at the nuclear region
glcm_corr_mean:DNA:dna_region	Mean correlation of DNA GLCM at the nuclear region
glcm_ent_mean:Actin:cell_region	Mean entropy of the actin GLCM at the whole-cell region
glcm_ent_mean:DNA:dna_region	Mean entropy of the DNA GLCM at the nuclear region
glcm_info_corr2_std:rH2AX:cell_region	Standard deviation of the information measure of correlation 2 of γH2AX GLCM at the whole-cell region
glcm_sum_ave_mean:gH2AX:cell_region	Mean sum average of γH2AX GLCM at the whole-cell region
glcm_sum_ent_mean:Actin:cell_region	Mean sum entropy of the actin GLCM at the whole-cell region
glcm_sum_var_std:DNA:dna_region	Standard deviation of the sum variance of DNA GLCM at the nuclear region
total_intensity:Actin:nondna_inner	Total actin intensity level at the inner cytoplasmic region
total_intensity_ratio:gH2AX-DNA:cell_region-cell_region	Ratio of the total γH2AX to DNA intensities at the whole-cell region
total_intensity_ratio:gH2AX-gH2AX:dna_region-cell_region	Ratio of the total γH2AX intensity levels at the nuclear region to the whole-cell region

Table S3. Average test accuracies for individual compounds.

Drug name	PTC Toxicity	HPTC-A (Single)	HPTC-A (Multi)	HPTC-B (Single)	HPTC-B (Multi)	HK-2 (Single)	HK-2 (Multi)
Lead(IV) acetate	Toxic	0%	80%	NA	NA	NA	NA
Aristolochic acid	Toxic	100%	100%	100%	100%	100%	100%
Arsenic(III) oxide	Toxic	100%	100%	100%	100%	100%	100%
Cadmium(II) chloride	Toxic	100%	100%	100%	100%	100%	100%
Cephalosporin C	Toxic	100%	100%	100%	100%	100%	100%
Cephalothin	Toxic	100%	100%	100%	100%	100%	100%
Citrinin	Toxic	100%	100%	100%	100%	100%	100%
Gold(I) chloride	Toxic	100%	100%	100%	100%	100%	100%
Tacrolimus	Toxic	10%	100%	100%	100%	100%	100%
Copper(II) chloride	Toxic	0%	20%	100%	100%	100%	100%
Rifampicin	Toxic	0%	100%	90%	100%	100%	100%
Puromycin	Toxic	90%	0%	0%	100%	100%	100%
Cephaloridine	Toxic	100%	0%	20%	90%	100%	100%
Bismuth(III) oxide	Toxic	100%	100%	0%	90%	100%	100%
Tenofovir	Toxic	80%	0%	100%	20%	100%	100%
Cisplatin	Toxic	100%	100%	100%	0%	100%	100%
Gentamicin	Toxic	90%	0%	0%	100%	90%	100%
Cyclosporin A	Toxic	100%	40%	0%	80%	90%	100%
Ochratoxin A	Toxic	100%	100%	100%	100%	20%	100%
Tetracycline	Toxic	100%	100%	100%	100%	0%	100%
Paraquat	Toxic	90%	100%	100%	100%	0%	100%
Potassium dichromate	Toxic	100%	100%	100%	80%	100%	90%
5-Fluorouracil	Toxic	100%	100%	20%	40%	0%	90%
Germanium(IV) oxide	Toxic	100%	90%	0%	10%	0%	90%
Hydrocortisone	Non-toxic	30%	90%	NA	NA	NA	NA
Acarbose	Non-toxic	100%	100%	100%	100%	100%	100%
Ethylene glycol	Non-toxic	100%	100%	100%	100%	100%	100%
Glycine	Non-toxic	100%	100%	100%	100%	100%	100%
Lincomycin	Non-toxic	100%	100%	100%	100%	100%	100%
Lindane	Non-toxic	100%	100%	100%	100%	100%	100%
Phenacetin	Non-toxic	100%	100%	100%	100%	100%	100%
Furosemide	Non-toxic	0%	100%	100%	100%	100%	100%
Dexamethasone	Non-toxic	100%	90%	100%	100%	100%	100%
Valacyclovir	Non-toxic	100%	90%	100%	100%	100%	100%
Metformin hydrochloride	Non-toxic	90%	50%	100%	100%	100%	100%
Melatonin	Non-toxic	100%	90%	10%	100%	100%	100%
Lithium chloride	Non-toxic	0%	90%	100%	90%	100%	100%
Ciprofloxacin	Non-toxic	0%	0%	0%	0%	100%	100%
Ibuprofen	Non-toxic	100%	100%	100%	100%	0%	100%
Triiodothyronine	Non-toxic	100%	100%	100%	100%	0%	90%
Levodopa	Non-toxic	0%	0%	30%	10%	100%	0%
Vancomycin	Non-toxic	100%	100%	100%	0%	100%	0%
Ribavirin	Non-toxic	90%	0%	30%	0%	100%	0%
Acetaminophen	Non-toxic	0%	100%	100%	100%	90%	0%

Supplementary Text S1

Algorithm S1. Classification performance estimation procedure

- Do for trial $i =$ 1 to 10:
  - Randomly divide the data into 10 folds with roughly equal numbers of compounds
  - Do for fold $j =$ 1 to 10:
    1. **Data division:** Assign the *j*-th fold to the final test set, $\mathbf{X}_{test}(\mathbf{F}_{all})$; and the other 7, 1, and 1 fold of compounds to $\mathbf{X}_{training}(\mathbf{F}_{all})$, $\mathbf{X}_{FStest}(\mathbf{F}_{all})$, $\mathbf{X}_{RFtest}(\mathbf{F}_{all})$, respectively
    2. **Feature selection:**
       - Compute the normalization coefficients based on $\mathbf{X}_{training}(\mathbf{F}_{all})$ and $\mathbf{X}_{FStest}(\mathbf{F}_{all})$, and normalize all datasets to obtain $\overline{\mathbf{X}}_{training}(\mathbf{F}_{all})$, $\overline{\mathbf{X}}_{FStest}(\mathbf{F}_{all})$, and $\overline{\mathbf{X}}_{RFtest}(\mathbf{F}_{all})$
       - Perform recursive feature elimination using $\overline{\mathbf{X}}_{training}(\mathbf{F}_{all})$, $\overline{\mathbf{X}}_{FStest}(\mathbf{F}_{all})$, and $\overline{\mathbf{X}}_{RFtest}(\mathbf{F}_{all})$ to obtain an optimum feature subset $\mathbf{F}_s$ (see **Algorithm S2**)
    3. **Feature importance estimation:**
       - Compute the normalization coefficients based on $\mathbf{X}_{training}(\mathbf{F}_s)$, $\mathbf{X}_{FStest}(\mathbf{F}_s)$ and $\mathbf{X}_{RFtest}(\mathbf{F}_s)$, and normalize all datasets to obtain $\overline{\mathbf{X}}_{training}(\mathbf{F}_s)$, $\overline{\mathbf{X}}_{FStest}(\mathbf{F}_s)$, $\overline{\mathbf{X}}_{RFtest}(\mathbf{F}_s)$, and $\overline{\mathbf{X}}_{test}(\mathbf{F}_s)$
       - Optimize N_{tree} and N_{trial} of a random forest using $\{\overline{\mathbf{X}}_{training}(\mathbf{F}_s), \overline{\mathbf{X}}_{FStest}(\mathbf{F}_s)\}$ as the training dataset, and $\overline{\mathbf{X}}_{RFtest}(\mathbf{F}_s)$ as the test dataset
       - Train a final random forest using the optimized N_{tree} and N_{trial} and $\{\overline{\mathbf{X}}_{training}(\mathbf{F}_s), \overline{\mathbf{X}}_{FStest}(\mathbf{F}_s), \overline{\mathbf{X}}_{RFtest}(\mathbf{F}_s)\}$ as the training dataset
       - Extract the importance values $\mathbf{w}(i, j)$ for all the features in $\mathbf{F}_s$
  - End do
- End do
- Set $\mathbf{F}_{final} \leftarrow$ features with the highest average $\mathbf{w}(i, j)$ values
- Do for trial $i =$ 1 to 10:
  - Do for fold $j =$ 1 to 10:
    4. **Final performance estimation:**
       - Divide the compounds into the same training and test sets as the previous loop
       - Repeat Step 3, but substitute $\mathbf{F}_s$ with $\mathbf{F}_{final}$
       - Determine the test accuracy of the final classifier in classifying $\overline{\mathbf{X}}_{test}(\mathbf{F}_{final})$
  - End do
- End do
- Compute the average test accuracy and $\mathbf{w}(i, j)$ across all the trials and folds

Supplementary Text S1 (continued)

Algorithm S2. Recursive feature elimination algorithm

Input:

- $\mathbf{F}_{all} = \{\mathbf{f}_1, \mathbf{f}_2, \cdots, \mathbf{f}_{m_{all}}\}$ (the set of all m_{all} extracted features)
- $\overline{\mathbf{X}}_{training}(\mathbf{F}_{all})$, $\overline{\mathbf{X}}_{FStest}(\mathbf{F}_{all})$, and $\overline{\mathbf{X}}_{RFtest}(\mathbf{F}_{all})$ (normalized training and test datasets based on all the extracted features, and there is no overlap between any pair of the datasets)

Output:

- $\mathbf{F}_s$ (the final m_s selected features)

1. Do for $j = m_{all}$ to 1 in m_Δ decrements:
   - Set the current training and test data $\overline{\mathbf{X}}'_{training} \leftarrow \overline{\mathbf{X}}_{training}(\mathbf{F}_j)$, $\overline{\mathbf{X}}'_{FStest} \leftarrow \overline{\mathbf{X}}_{FStest}(\mathbf{F}_j)$, and $\overline{\mathbf{X}}'_{RFtest} \leftarrow \overline{\mathbf{X}}_{RFtest}(\mathbf{F}_j)$
   - Optimize the parameters N_{tree} and N_{trial} of a random forest RF_{temp} using $\overline{\mathbf{X}}'_{training}$ as the training dataset and $\overline{\mathbf{X}}'_{FStest}$ as the test dataset
   - Trained a final random forest RF_{final} using the optimized parameters and both $\{\overline{\mathbf{X}}'_{training}, \overline{\mathbf{X}}'_{FStest}\}$ as the training dataset
   - Set acc_j to be the balanced test accuracy of RF_{final} in classifying $\overline{\mathbf{X}}'_{RFtest}$
   - If $j > 1$
     1. Extract the importance values of all the features in $\mathbf{F}_j$ from RF_{final}
     2. Identify a set of m_Δ features, $\mathbf{F}_{lowest} \subseteq \mathbf{F}_j$, with the lowest importance values
     3. Update the feature list $\mathbf{F}_{j-1} \leftarrow \mathbf{F}_j - \mathbf{F}_{lowest}$

   End do
2. Model all the acc_j values as a mixture of Gaussian distributions:
   - Fit a smoothing spline $g_{acc}(j)$ to all the acc_j values
   - Identify the local maximum $\hat{g}_{acc}$ of $g_{acc}(j)$ with the smallest j
   - Set the mean of the first Gaussian distribution to $\hat{g}_{acc}$
   - Perform Gaussian mixture modeling for the number of mixtures, k = 2 to 4
   - Determine the optimum k based on the Bayes Information Criterion (BIC)
   - Determine the 5%-tile and 95%-tile of the first Gaussian distribution
3. Select the feature subset $\mathbf{F}_s$ with the smallest j and acc_j within the percentile range
